# Supplementary material for: Influence of short and long term processes on SAR11 communities in open ocean and coastal systems
Source: ISME Commun. 2022 Nov 19;2:116. doi: 10.1038/s43705-022-00198-1 (PMC9723719; doi:10.1038/s43705-022-00198-1)
Supplement: Supplementary file 1 — Supplementary Material [file 43705_2022_198_MOESM1_ESM.docx]

Influence of short and long term processes on SAR11 communities in open ocean and coastal systems

Luis M. Bolaños^1*^, Karen Tait^2^, Paul J. Somerfield^2^, Rachel J. Parsons^3^, Stephen J. Giovannoni^4^, Timothy Smyth^2^, Ben Temperton^1*^

1 School of Biosciences, University of Exeter, Exeter, UK

2 Plymouth Marine Laboratory, Plymouth, UK

3 Bermuda Institute of Ocean Sciences, St. George’s, Bermuda

4 Department of Microbiology, Oregon State University, Corvallis, OR, USA

***Corresponding Author**

l.bolanos-avellaneda@exeter.ac.uk

b.temperton@exeter.ac.uk

**Supplementary Information**

**Supporting Methods**

**Bioinformatics analysis:**

Bermuda Atlantic Time Series

454 FLX (1991-2002) sequence processing

Raw ssf data files were demultiplexed using get.mimarkspackage command from Mothur (1). Fasta and qual files were generated from the individual samples using sffinfo command. Fastq files for each pair of fasta and quality files were generated with the make.fastq. Fastq files were trimmed with CutAdapt (-u 28). Adapter-trimmed sequences were used to generate ASV count table with Dada2 (2). Sequences were quality filtered using the following parameters maxEE=4, rm.phix=TRUE, minLen=190, truncLen= 220. Sequences were denoised to control for homopolymer artifacts. Samples were processed by plates to accurately estimate the error frequency. Taxonomic assignment was performed with the assignTaxonomy command and the silva non redundant database V123 (3). Finally, an ASV table was generated for each plate. ASV tables were merged using merge_phyloseq command in Phyloseq R package (4).

Illumina MiSeq (2015-2018) sequence processing.

Primers from the demultiplexed raw paired-end fastq files were cut using the CutAdapt (5), removing 20 bases from forward files and 18 from reverse files that matched the 27F and 338 RPL primer lengths, respectively. Sequences were quality filtered using the following parameters maxEE=(2,2), truncQ=2, minLen=190, truncLen= 220,190, maxN=0. Quality filtered sequences were dereplicated and merged with dada2 R package, version 1.2 (2). Sequences from the same run were processed together to accurately estimate the error frequency. Taxonomic assignment was performed with the assignTaxonomy command and the silva non redundant database V123 (3). ASV tables were generated for each plate. ASV tables were merged using merge_phyloseq command in Phyloseq R package (4).

SAR11 ASVs taxonomic assignment

SAR11 ASV tables were obtained by sub setting the sequences assigned to the order "SAR11_clade". SAR11 sequences from the 454 FLX and MiSeq datasets were aligned using mafft (Katoh et al., 2002). The alignment were manually trimmed and the resulting file was collapsed to dereplicate potential duplicated ASVs generated after trimming. The ASV counts were collapsed accordingly. Finally, a fasta file of the unique ASVs were generated. SAR11 sequences were placed in a curated reference tree (6) using Phyloassigner version v6.166 (7). The result of the highly-resolved phylogenetic placement was used to update the taxonomic file.

Western Channel Observatory (2012-2018)

Illumina MiSeq sequence processing.

Primers from the demultiplexed raw paired-end fastq files were cut using the CutAdapt (5), removing the primer sequences based in all potential orientations. Sequences were quality filtered using the following parameters maxEE=(2,2), truncQ=2, truncLen= (240,200), maxN=0. Quality filtered sequences were dereplicated and merged with dada2 R package, version 1.2 (2). Sequences from the same run were processed together to accurately estimate the error frequency. Taxonomic assignment was performed with the assignTaxonomy command and the silva non redundant database V138 (3). ASV tables were merged using merge_phyloseq command in Phyloseq R package (4).

SAR11 ASVs taxonomic assignment

SAR11 ASV tables were obtained by sub setting the sequences assigned to the order "SAR11_clade". The SAR11 ASVs were placed in a curated reference tree (6) using Phyloassigner version v6.166 (7). The result of the highly-resolved phylogenetic placement was used to generate an updated taxonomic file.

**Supporting Results**

**SAR11 ecotype seasonality is persistent through multiannual time-series in the Sargasso Sea and Western English Channel**

SAR11 fraction was comprised of 557 ASVs (454 FLX) and 697 (MiSeq) at BATS and 168 at WEC before filtering by minimum abundance (>.0015). From these, 518, 650 and 147 were assigned to terminal nodes of the SAR11 phylogenetic tree, respectively (Fig S3). For this study, we considered the full internal and terminal nodes collection of SAR11 ASVs.

We evaluated whether the SAR11 ASVs from the BATS period of 2016-2018 with relative abundance > 0.05% presented a significant periodicity (Fisher *G*-test, p < 0.05). From the top 169 ASVs, 49.7% displayed a significant periodicity through the analysed years (representing 65.02% of the total reads). From the 11 most abundant ASVs (>2%), five (45.45%) showed a significant periodicity (Fig. S1). Similarly, we evaluated the periodicity of SAR11 ASVs retrieved at the WEC. We analysed the total 144 WEC ASVs, from which 88.2% were determined to display significant periodicity (representing 99.4% of the total reads), including the 10 most abundant ASVs (>2%) (Fig. S2). SAR11 experiencing seasonal periodicity is much higher in WEC than BATS, most likely due to the much bigger seasonal shifts or the V4-V5 region higher conservation (Fig. S12) masking minority ASVs dynamics.

**Supporting References**

1. Schloss PD, Westcott SL, Ryabin T, Hall JR, Hartmann M, Hollister EB, et al. Introducing mothur: Open-Source, Platform-Independent, Community-Supported Software for Describing and Comparing Microbial Communities. Appl Environ Microbiol. 2009;75:7537–41.

2. Callahan BJ, McMurdie PJ, Rosen MJ, Han AW, Johnson AJA, Holmes SP. DADA2: High-resolution sample inference from Illumina amplicon data. Nat Methods. 2016;13:581–3.

3. Quast C, Pruesse E, Yilmaz P, Gerken J, Schweer T, Yarza P, et al. The SILVA ribosomal RNA gene database project: improved data processing and web-based tools. Nucleic Acids Res. 2012;41:D590–6.

4. McMurdie PJ, Holmes S. phyloseq: An R Package for Reproducible Interactive Analysis and Graphics of Microbiome Census Data. Watson M, editor. PLoS ONE. 2013 Apr 22;8(4):e61217.

5. Martin M. Cutadapt removes adapter sequences from high-throughput sequencing reads. EMBnet.journal. 2011;17:10.

6. Bolaños LM, Choi CJ, Worden AZ, Baetge N, Carlson CA, Giovannoni S. Seasonality of the Microbial Community Composition in the North Atlantic. Front Mar Sci. 2021;8:624164.

7. Vergin KL, Beszteri B, Monier A, Cameron Thrash J, Temperton B, Treusch AH, et al. High-resolution SAR11 ecotype dynamics at the Bermuda Atlantic Time-series Study site by phylogenetic placement of pyrosequences. ISME J. 2013;7:1322–32.

**Supplementary Tables and Figures**

**Supplementary Table 1. Time series samples used in this study and their corresponding environmental data (Provided as an independent document).**


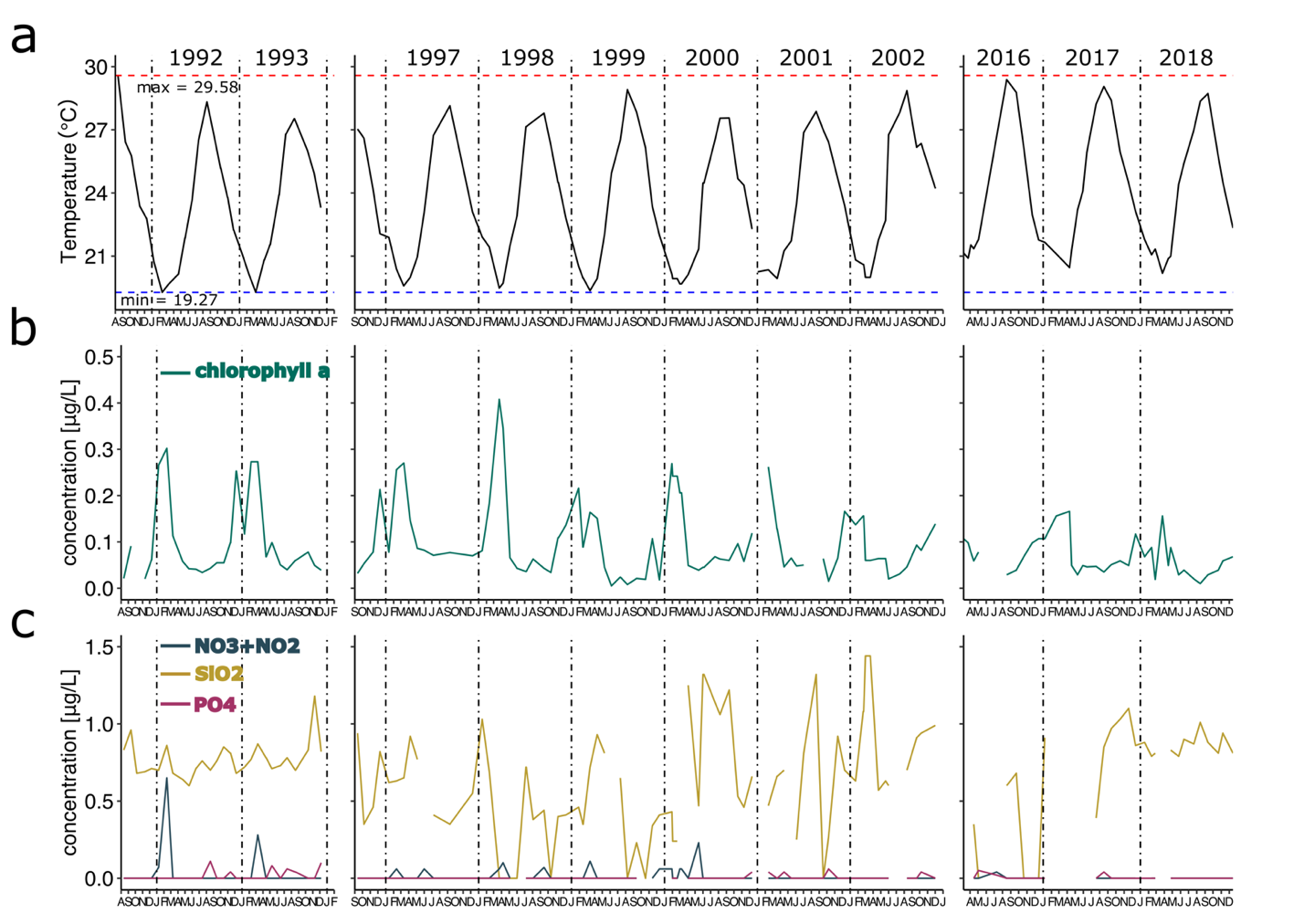


**Supplementary Figure 1. Surface (5m) physicochemical environmental variables measured in the Bermuda Atlantic Time-series Study from 1991 to 2018.** The discontinuity of the lines represents missing values. **(a) Temperature**. The maximum temperature from the measured time ranges is shown as a red dashed line. The minimum temperature is shown as a blue dashed line. **(b) Chlorophyll a**. Low chlorophyll concentrations (<0.25 ug/L) were present for most of the time-range, except for early and late 90s winter to spring transitions. A long-term decreasing trend in chlorophyll a concentration from the early 90s to late 2010s is evident **(c) Nutrients.** Nitrate and nitrite (blue) and phosphate (pink) were close to the detection limits in most of the samples. Silica (yellow) displayed variable concentrations throughout the sampled years without a distinguishable pattern.


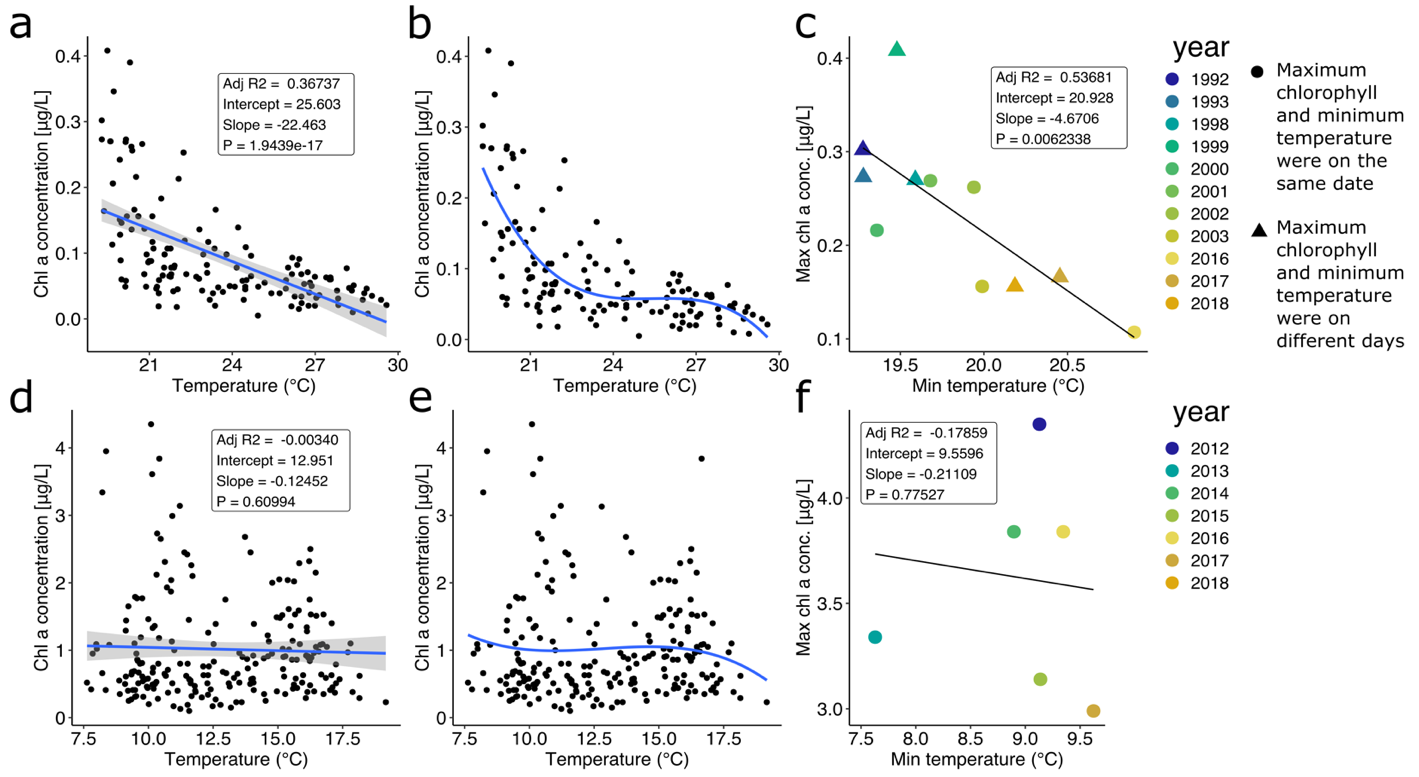


**Supplementary Figure 2. Correlation of chlorophyll a concentrations and temperature of BATS and WEC time-series. (a)** BATS linear regression of concurrent measurements of chlorophyll and temperature. **(b)** BATS polynomial regression of concurrent measurements of chlorophyll and temperature. **(c)** BATS linear regression of the maximum chlorophyll concentration and the minimum temperature for each year sampled during the time-series. **(d)** WEC linear regression of concurrent measurements of chlorophyll and temperature. **(e)** WEC polynomial regression of concurrent measurements of chlorophyll and temperature. **(f)** WEC linear regression of the maximum chlorophyll concentration and the minimum temperature for each year sampled during the time-series. In **(c)** and **(f)** shape represent whether the maximum chlorophyll concentration and minimum temperature measurements belong from the same sampling day (circle) or in a different day (triangle).

**
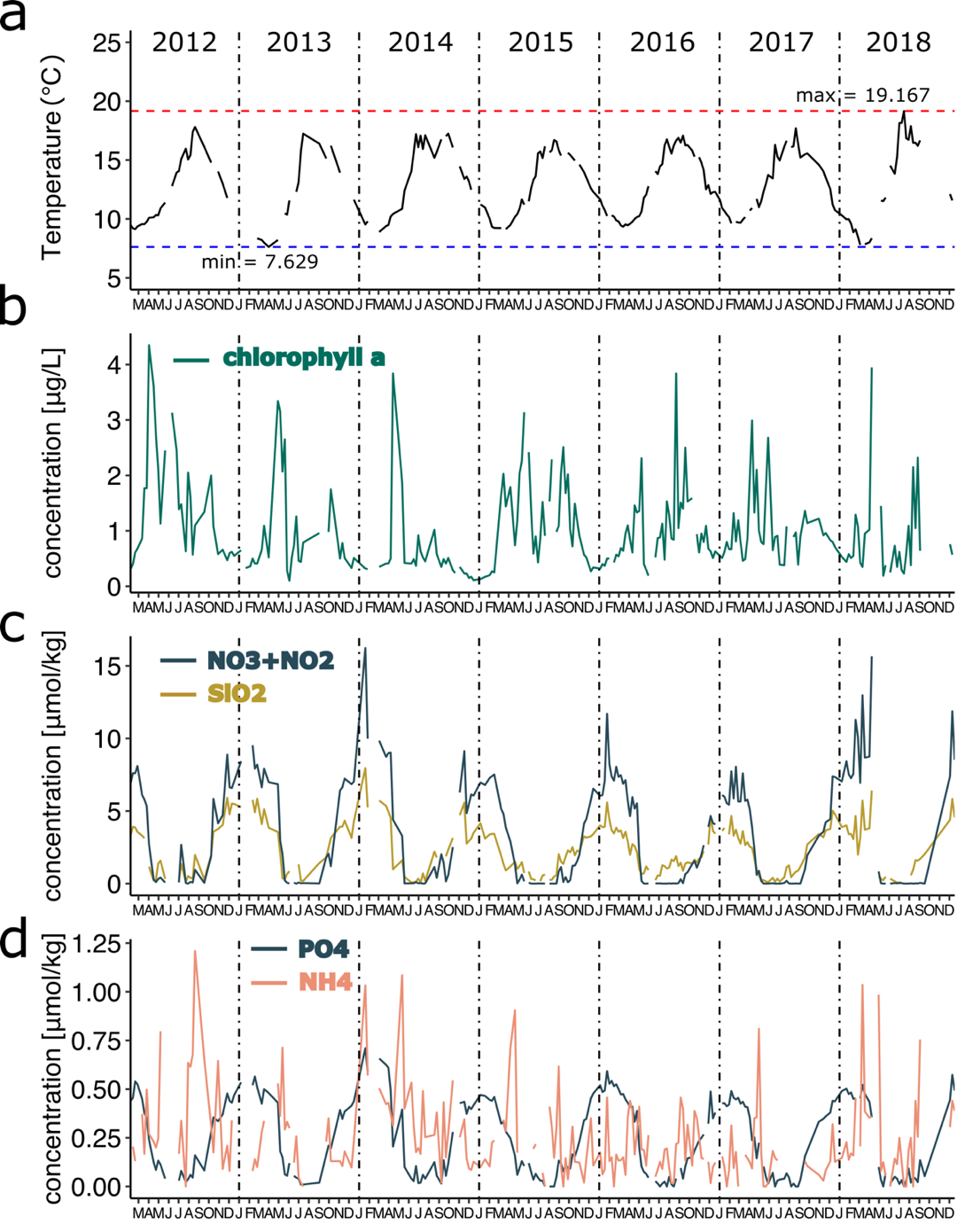
**

**Supplementary Figure 3. Surface (5m) physicochemical environmental variables measured in the Western English Channel (station L4) from 2012 to 2018.** The discontinuity of the lines represents missing values. **(a) Temperature**. The maximum temperature from the measured time ranges is shown as a red dashed line. The minimum temperature is shown as a blue dashed line. **(b) Chlorophyll a** **concentration.** High concentration peaks display inter-annual variability; however, spring and autumn bloom peaks are consistent throughout the sampled years. **(c) Nutrients.** Nitrate and nitrite (blue) and silica (yellow) displayed a seasonal oscillation with high concentrations in autumn and winter, while being close to detection limits in spring and summer. **(d) Nutrients**. Phosphate (blue) in panel (d) displayed a seasonal oscillation similar to Nitrate and nitrite (blue) and silica (gold) in panel (c). Ammonium concentrations were highly variable within the range of 0 to 1.25 umol/Kg.


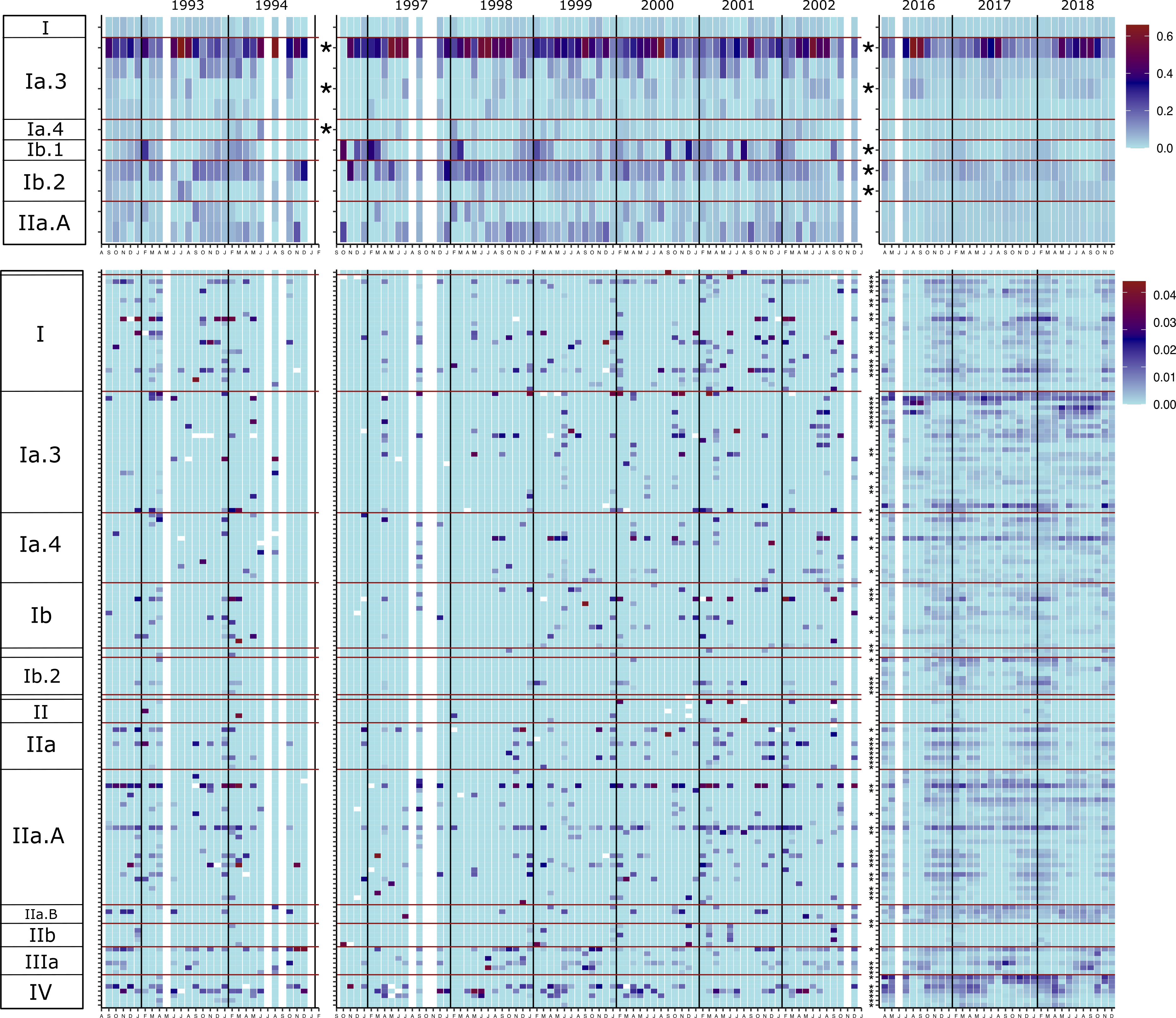


**Supplementary Figure 4. Heat map depicting the monthly relative abundance of the top 169 SAR11 ASVs from the Bermuda Atlantic Time-series Study.** ASVs with a relative abundance > 0.05% were selected. The ASVs are divided into two groups based on its contribution. The top panel displays ASVs with a relative contribution greater than 2% (top 11 ASVs). The bottom panel displays ASVs with a relative contribution less than 2% (158 ASVs). The ASVs are sorted in the y-axis by ecotype for both panels. Within ecotypes, ASVs are sorted vertically by decreasing relative contribution to the dataset. Asterisk in the y-axis indicates that the ASV displays a significant periodic pattern based on a Fisher G-test (p < 0.05) (See Supplementary Materials and Methods). Fisher G-test were done independently for the period between 1991-2002 and 2016-2018.


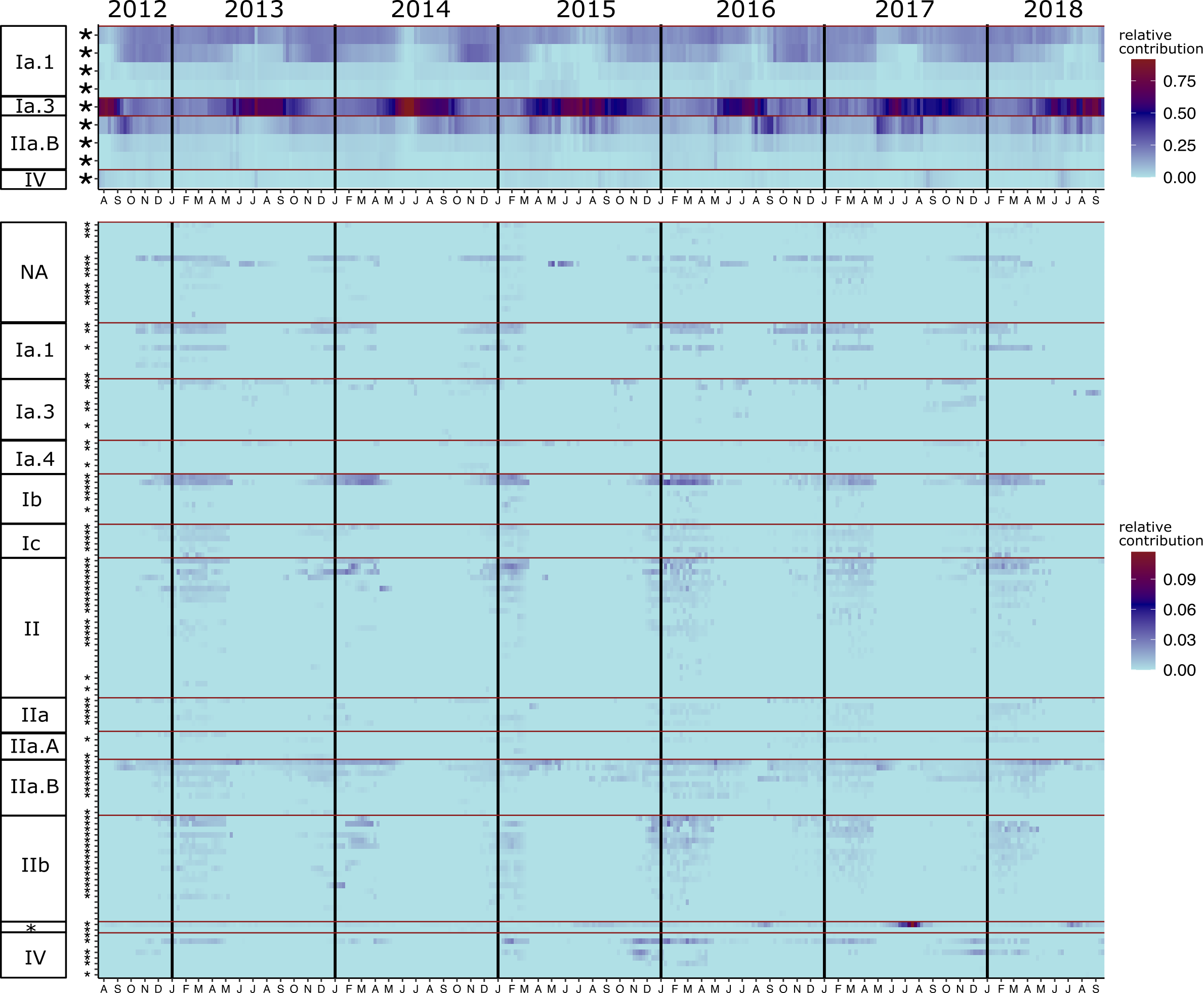


**Supplementary Figure 5. Heat map depicting the weekly relative abundance of the SAR11 ASVs from the Western English Channel.** All SAR11 ASVs (144) are depicted. Samples with less than 1000 SAR11 reads were removed. The missing weekly samples were interpolated using the final dataset. The ASVs are divided into two groups based on its contribution. The top panel displays ASVs with a relative contribution greater than 2% (top 9 ASVs). The bottom panel displays ASVs with a relative contribution less than 2% (158 ASVs). ASVs are sorted in the y-axis by ecotype for both panels. Within ecotypes, ASVs are sorted vertically by decreasing relative contribution to the dataset. Asterisk in the y-axis indicates that the ASV displays a significant periodic pattern (estimated in between 1^st^ September 2013 and 28^th^ August 2018) based on a Fisher G-test (p < 0.05) (See Supplementary Materials and Methods). [^X^] = clade N2.


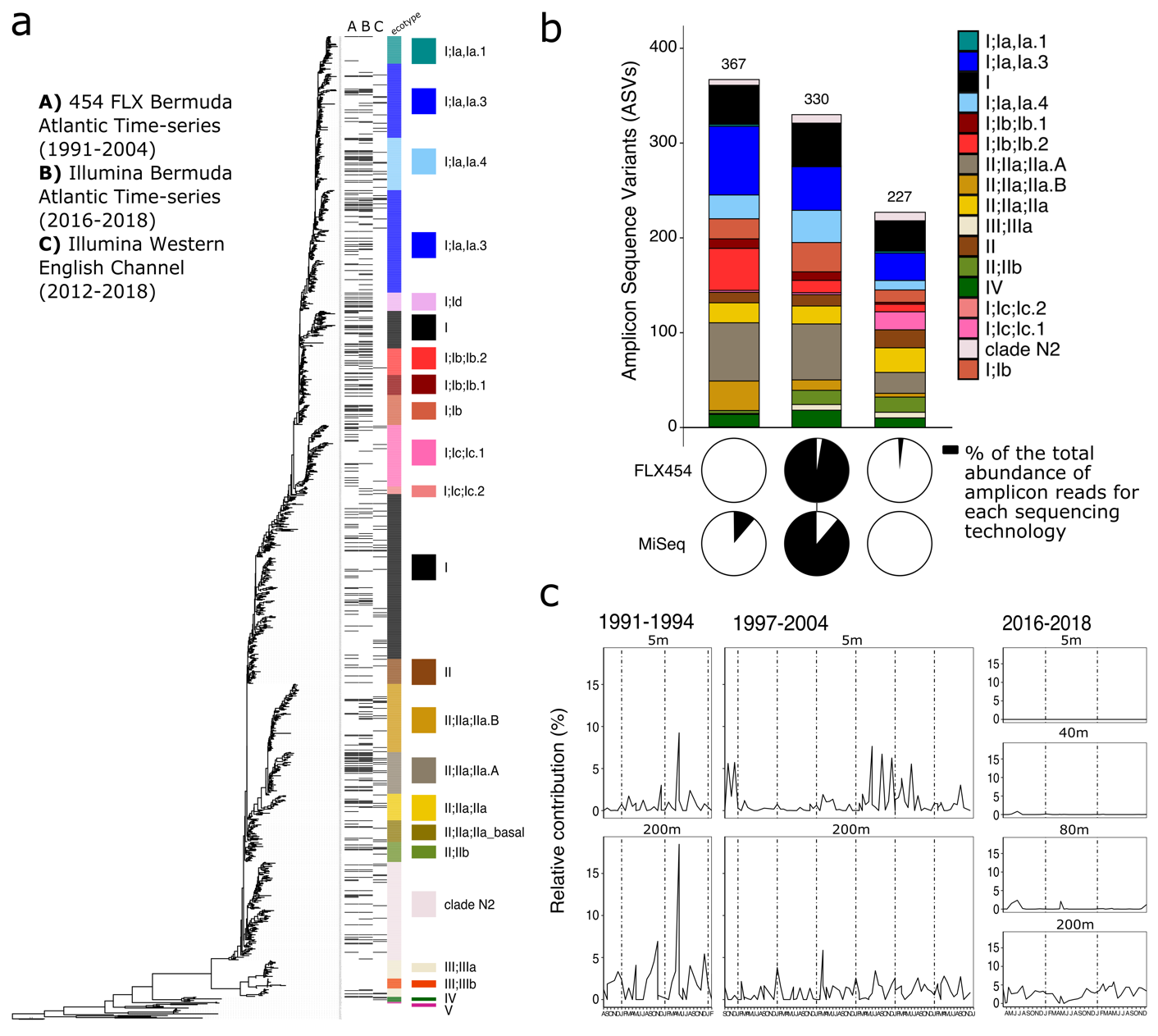


**Supplementary Figure 6. Phylogenetic assignment and distribution of SAR11 ASVs. (a)** Terminal node ASV profiles for the different datasets: BATS 454FLX, BATS MiSeq and WEC MiSeq. The full-length 16S rRNA phylogenetic tree used as database to assign the ASV sequences is depicted on the left (Bolaños et al., 2021). Three columns showing the presence (black) and absence (white) of ASVs for each terminal node are shown in front of the corresponding tip of the tree. Each column corresponds to a dataset (**A:** BATS 454FLX**, B:** BATS MiSeq and **C:** WEC MiSeq). The fourth column on the right side show a color-coded relational table for each tip on the tree to the ecotype it belongs. Ia.1 WEC sequences were placed in internal nodes due to its high conservation and therefore not shown in this figure. (b) Visualization of unique and common ASVs (intersection) between 454 FLX and MiSeq. Bar plots depict the taxonomic distribution of SAR11 ASVs within the groups (454 FLX unique, intersection and MiSeq unique). Pie diagrams depict the percentage of the total amplicon abundance represented by the ASVs in the bar plot. (c) Time series of the relative abundance of ASVs found exclusively in 454 FLX at different depths in the BATS dataset (454 FLX and MiSeq). Exclusive 454 FLX ASVs in the 5 mts samples were found in deeper samples (40, 80 and 200 m.) sequenced using MiSeq (2016-2018). This suggests that the absence of these ASVs in the surface samples from 2016-2018 is a real decrease or vanish and not an artifact caused by the sequencing platform.

**
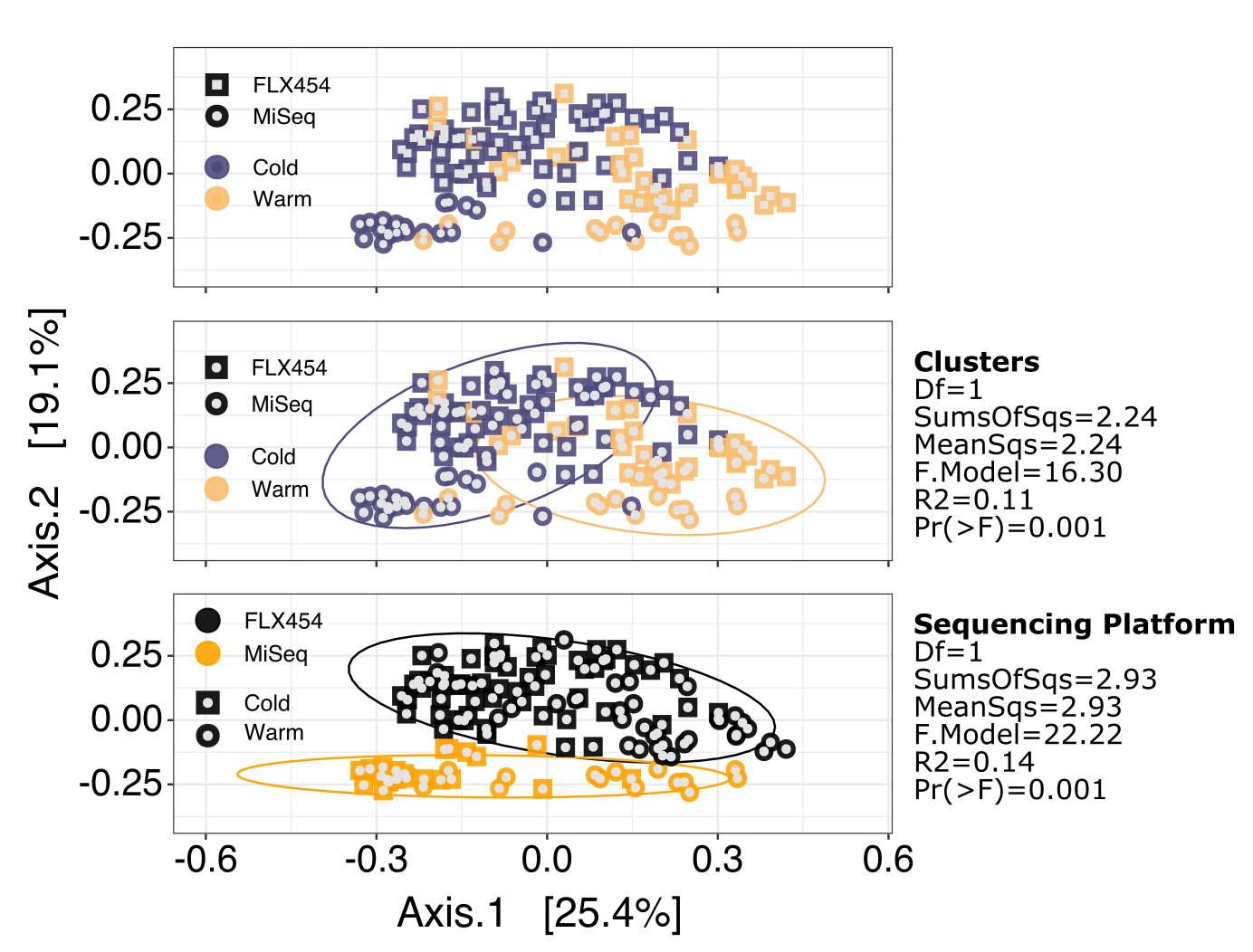
**

**Supplementary Figure 7. SAR11 multidimensional scaling (MDS) ordination based on Bray-Curtis dissimilarities.** Top panel represents the original MDS. Middle panel is the original ordination overlapped by 90% ellipses around the centroids of the sample category clusters. Both, top and middle panels, are color coded by “cluster” (cold-warm) category and shape coded by sequencing platform. Bottom panel represents the original MDS color coded by sequencing platform used to generate the amplicon datasets and 90% ellipses around the centroids of the sequencing platform category clusters. On the right side of the middle and bottom panels we present the Permutational Multivariate Analysis of Variance that support the differences between clusters and sequencing platform.

**Supplementary Figure 8. ASVs dendrograms depicting the hierarchical clustering of the samples based on the SAR11 ASV profiles at the Bermuda Atlantic Time-series Study. (a)** ASVs dendrogram defined by hierarchical clustering of the SAR11 community at 5 m depth color coded by seasons in the northern hemisphere. **(b)** ASVs dendrogram defined by hierarchical clustering of the SAR11 community at 5 m depth color coded by a broader definition of seasonality for SAR11 based on the dynamics presented in figures 4 and 5. “Cold” comprises samples from 22 September to 1^st^ May and “Warm” from 2^nd^ May to 21 September. Heatmaps (right) represent the temperature recorded for the sample and sequencing technology used to generate the amplicon data on the corresponding node for both dendrograms.

**Supplementary Figure 9. ASVs dendrogram depicting the hierarchical clustering of the samples based on the SAR11 ASV profiles at the Western English Channel (2012-2018). (a)** ASVs dendrogram defined by hierarchical clustering of the SAR11 community at 5 m depth color coded by seasons in the northern hemisphere. **(b)** ASVs dendrogram defined by hierarchical clustering of the SAR11 community at 5 m depth color coded by a broader definition of seasonality for SAR11 based on the dynamics presented in figures 4 and 5. “Cold” comprises samples from 22 September to 1^st^ May and “Warm” from 2^nd^ May to 21 September. Heatmap (right) represents the temperature recorded for the sample on the corresponding node for both dendrograms.

**
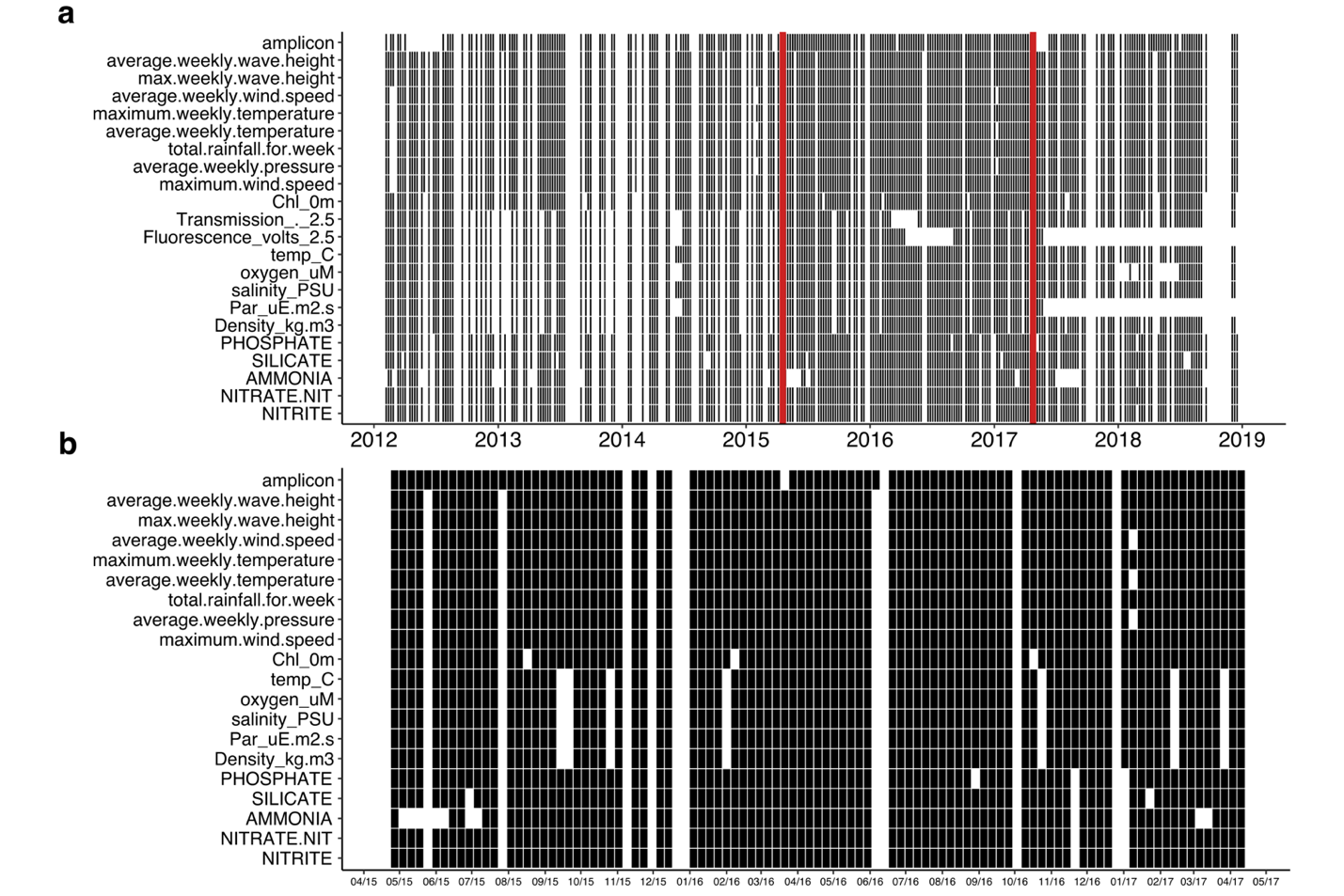
**

**Supplementary Figure 10. Binary representation of the presence and absence of amplicon datasets and their corresponding environmental measurements at the Western English Channel from 2012 to 2018.** Black squares represent the presence of the indicated measurement, while whites represent the absence. **(a)** Representation of the collected measurements throughout the complete time-series analysed in this study **(b)** Fraction of the measurements taken between April 2015 and April 2017, indicated between red lines in panel (a).

**
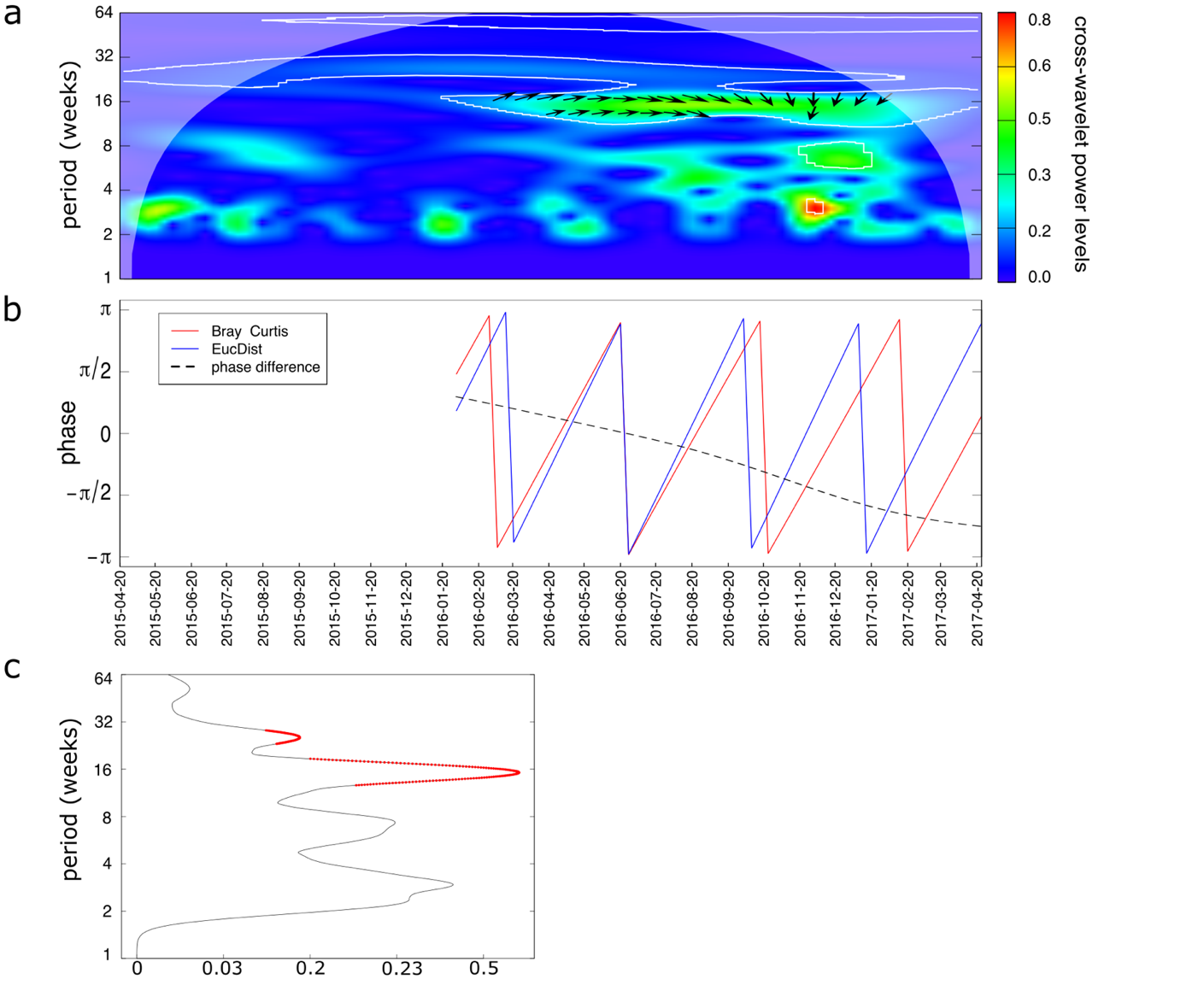
**

**Supplementary Figure 11.** **Wavelet coherence analysis between Bray–Curtis dissimilarity and Euclidean distances in the period between April 2015 and April 2017 at the Western English Channel. (a)** Cross-wavelet power levels represented as a color gradient. Periods with significant coherence (P > 0.05) are illustrated by a white line. The angle of the arrows indicate whether the two variables are in phase (positive correlation) and its direction if these are lagged. In phase with x followed by y corresponds to a 45 degree angle (↗), out of phase with y followed by x corresponds to a 135 degree angle (↘). In phase and out of phase without lagging periods are represented by → and ←, respectively. **(b)** In phase representation of the two variables (red and blue) overlapped by the phase difference progression (dashed black line). **(c)** Average coherence for the significant in phase period (P > 0.05) is indicated in red.

**
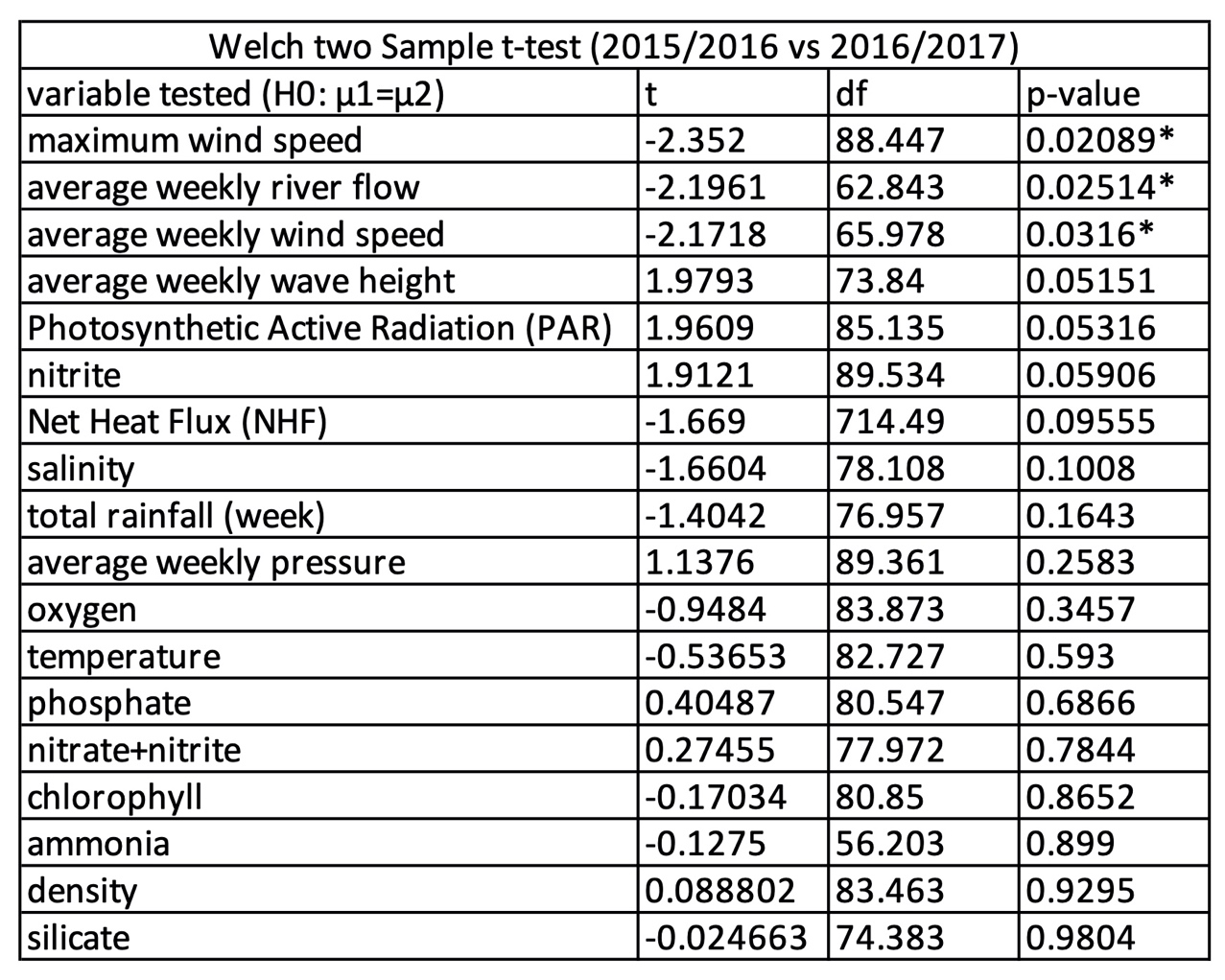
**

**Supplementary Table 2. Welch's two sample t-test results comparing coupled and uncoupled measurements between April 2015 and April 2017 at the Western English Channel.** * p-value < 0.05.

**
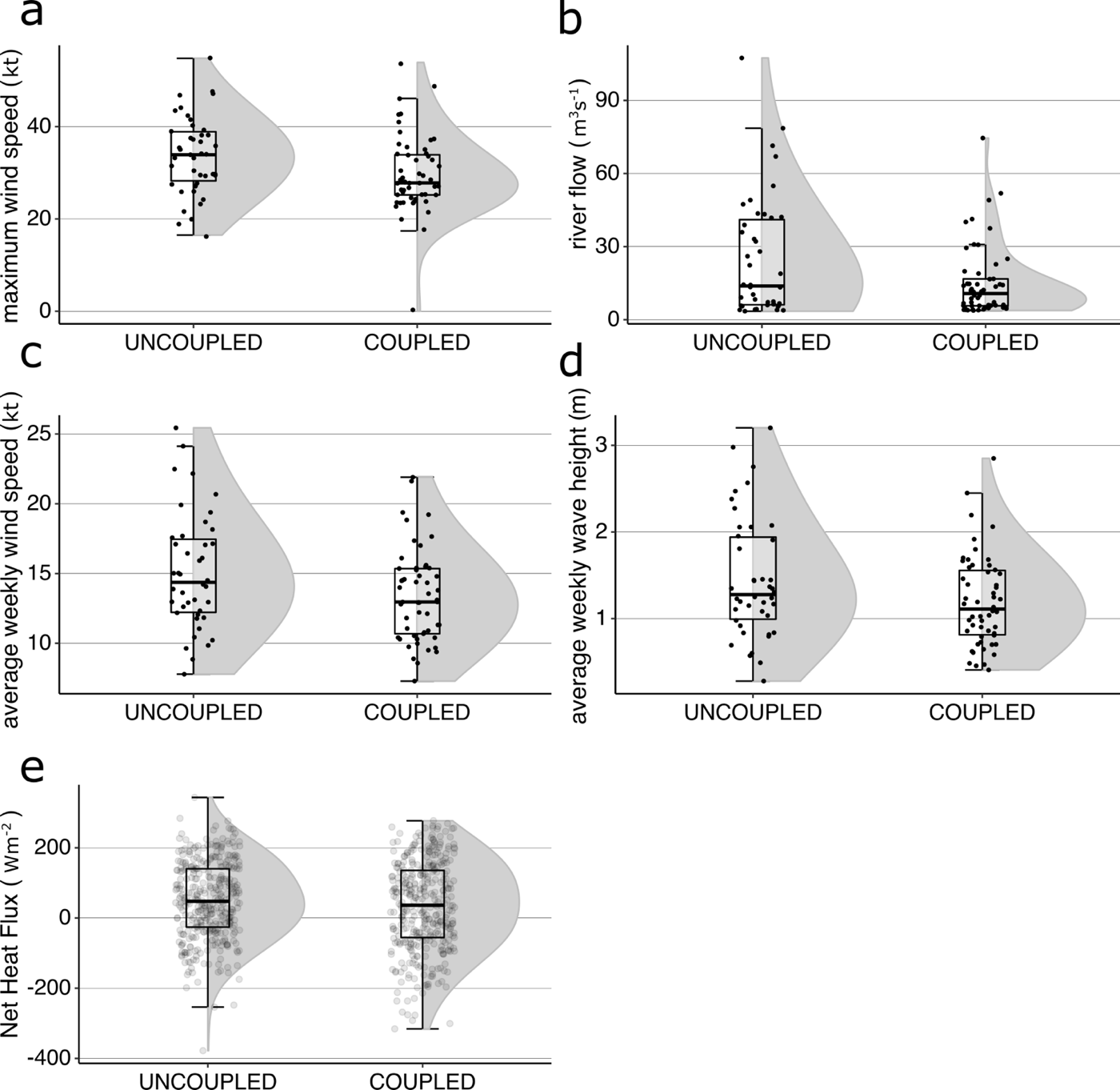
**

**Supplementary Figure 12. Violin plot overlapped by a boxplot and the corresponding measurements as black points of the variables that showed a significant difference (a,b,c) during the uncoupled and coupled periods from April 2015 to April 2017 at the Western English Channel. Average weekly wave height (d) and Net Heat Flux (e) are also shown for comparison.**

**
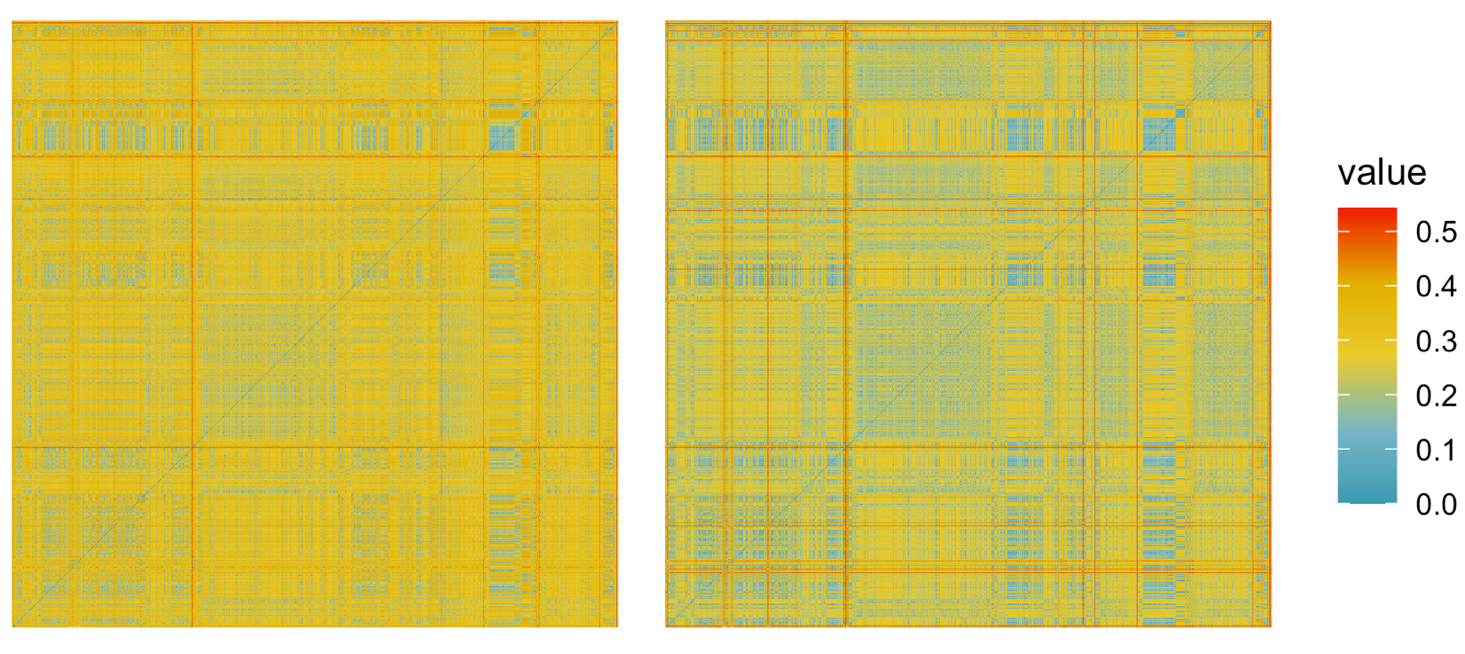
**

**Supplementary Figure 13. Heatmap matrix depicting the pairwise genetic distance of the V1-V2 and V4-V5 hypervariable regions between the full length sequences used to reconstruct the SAR11 phylogenetic database (Fig S6, Bolaños et al., 2021).** Genetic distances were estimated on the fragments excised from the original alignment using dist.alignment command from the seqinr (Charif D, Lobry J 2007) R package. V1-V2 region is shown on the left panel and V4-V5 on the right. The color-code ranges from identical sequences (0 distance, blue) to 50% identity (0.5 distance, red). The pairwise distances of V1-V2 and V4-V5 are significantly different (Welsch t = 532.88, df = 2683441, p-value < 2.2e-16), supporting that the level of conservation of V4-V5 is higher than the V1-V2.

**
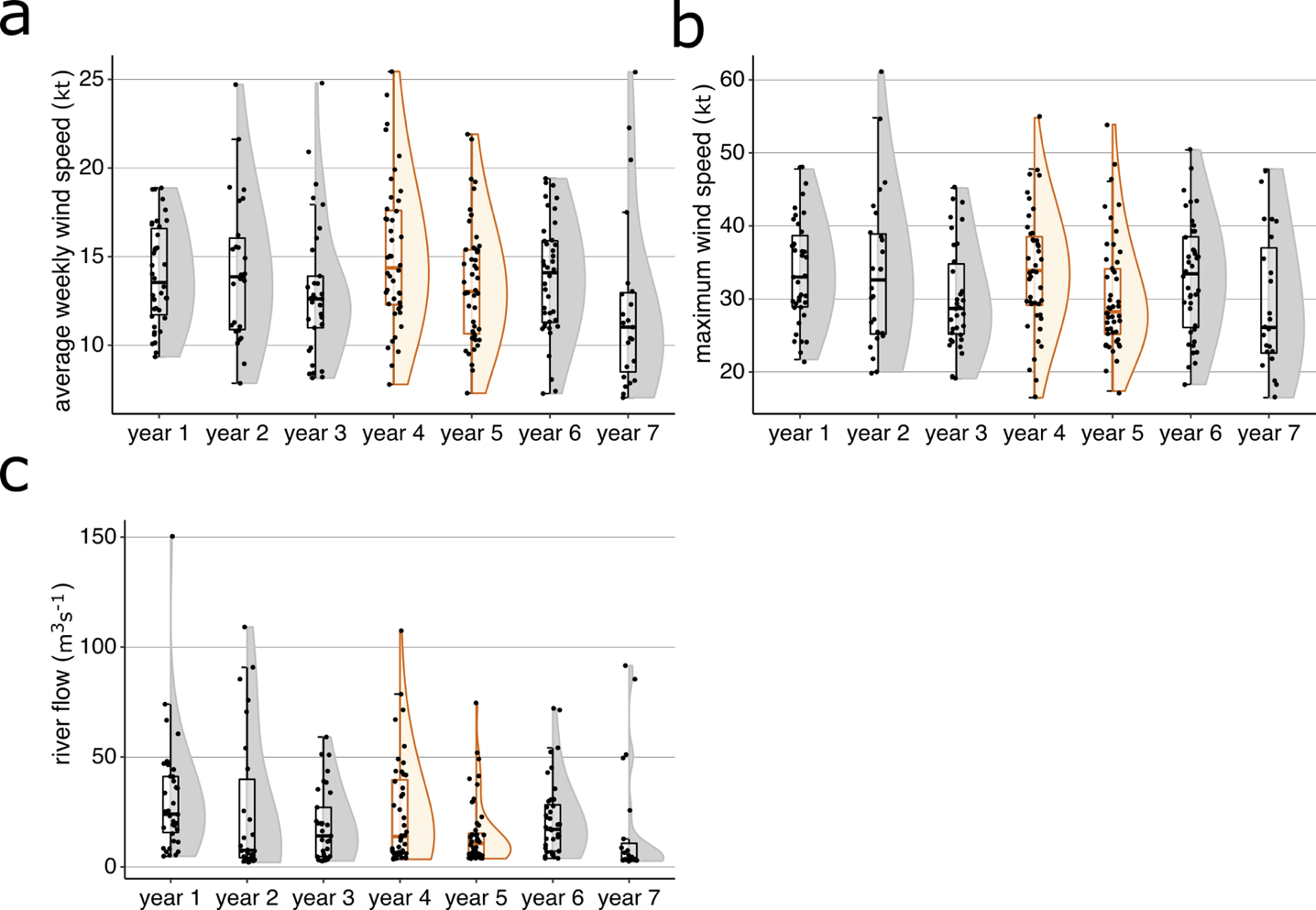
**

**Supplementary Figure 14. Distribution of average weekly wind speed, maximum wind speed and river flow from 2012 to 2018 at the Western English Channel. Violin plot overlapped by a boxplot and the corresponding measurements as black points.** Each group labeled as “year” in the x-axis represent a year starting and ending in the vernal equinox (March 21). Year 4 (2015-2016) and year 5 (2016-2017) are highlighted in yellow. These years correspond to the highly weekly sampling period showed in Fig S10 (between red lines in panel ‘a’ and panel ‘b’).
