# Supplementary material for: Influence of short and long term processes on SAR11 communities in open ocean and coastal systems
Source: ISME Commun. 2022 Nov 19;2:116. doi: 10.1038/s43705-022-00198-1 (PMC9723719; doi:10.1038/s43705-022-00198-1)
Supplement: Supplementary file 2 — Supplementary Table 1 [file 43705_2022_198_MOESM2_ESM.pdf]

Bermuda Atlantic Time-series												
Date (YYYY-MM-DD)	Sample	Temperature (°C)	Salinity (p.s.u.)	O2 (µmol/kg)	NO2+NO3 (µmol/kg)	NO3 (µmol/kg)	NO2 (µmol/kg)	PO4 (µmol/kg)	SiO2 (µmol/kg)	Bacteria (cells*10^8/kg)	Turner. Chl.a (µg/L)	Reference
1991-08-12	35-0	29.580	36.893	195.830	0	0	0	0	0.83	1.6	0.02	Vergin et al., 2013
1991-09-12	36-0	26.420	36.266	206.390	0	0	0	0	0.96	4.01	0.09	Vergin et al., 2013
1991-10-07	37-0	25.770	36.496	210.820	0	0	0	0	0.68	4.31	-	Vergin et al., 2013
1991-11-11	38-0	23.380	36.598	217.090	0	0	0	0	0.69	4.38	0.02	Vergin et al., 2013
1991-12-09	39-0	22.796	36.804	219.930	0	0	0	0	0.71	4.52	0.06	Vergin et al., 2013
1992-01-09	40-0	20.742	36.697	-	0.07	0.03	0.04	0	0.7	4.81	0.27	Vergin et al., 2013
1992-02-13	41-0	19.277	36.677	224.720	0.65	0.36	0.29	0	0.86	5.91	0.3	Vergin et al., 2013
1992-03-10	42-0	19.659	36.659	229.170	0	0	0	0	0.68	7.29	0.11	Vergin et al., 2013
1992-04-21	43-0	20.150	36.786	226.760	0	0	0	0	0.64	4.46	0.06	Vergin et al., 2013
1992-05-18	44-0	21.867	36.710	223.750	0	0	0	0	0.6	-	0.04	Vergin et al., 2013
1992-06-17	45-0	23.661	36.398	215.770	0	0	0	0	0.71	6.43	0.04	Vergin et al., 2013
1992-07-14	46-0	26.503	36.370	206.860	0	0	0	0	0.76	4.6	0.03	Vergin et al., 2013
1992-08-18	47-0	28.327	36.449	201.890	0	0	0	0.11	0.7	4.37	0.04	Vergin et al., 2013
1992-09-15	48-0	26.810	36.299	202.780	0	0	0	0	0.76	7	0.06	Vergin et al., 2013
1992-10-14	49-0	25.172	36.340	207.130	0	0	0	0	0.85	-	0.06	Vergin et al., 2013
1992-11-12	50-0	23.788	36.540	211.220	0	0	0	0.04	0.81	4.93	0.1	Vergin et al., 2013
1992-12-07	51-0	22.235	36.578	215.100	0	0	0	0	0.68	4.9	0.25	Vergin et al., 2013
1993-01-12	52-0	21.110	36.660	222.230	0	0	0	0	0.72	4.68	0.12	Vergin et al., 2013
1993-02-09	53-0	20.153	36.670	217.400	0	0	0	0	0.77	-	0.27	Vergin et al., 2013
1993-03-10	54-0	19.279	36.640	224.190	0.28	0.19	0.088	0	0.87	4.76	0.27	Vergin et al., 2013
1993-04-14	55-0	20.815	36.750	-	0	0	0	0	0.78	-	0.07	Vergin et al., 2013
1993-05-09	56-0	21.550	36.542	226.410	0	0	0	0.08	0.71	5.82	0.1	Vergin et al., 2013
1993-06-15	57-0	23.910	36.761	209.710	0	0	0	0	0.73	4.56	0.05	Vergin et al., 2013
1993-07-13	58-0	26.790	36.745	205.700	0	0	0	0.06	0.78	5.2	0.04	Vergin et al., 2013
1993-08-18	59-0	27.524	36.667	200.300	0	0	0	0.04	0.7	4.25	0.06	Vergin et al., 2013
1993-10-12	61-0	26.020	36.435	204.130	0	0	0	0	0.83	4.82	0.08	Vergin et al., 2013
1993-11-09	62-0	24.940	36.526	209.400	0	0	0	0	1.18	5.23	0.05	Vergin et al., 2013
1993-12-07	63-0	23.300	36.476	213.500	0	0	0	0.1	0.82	3.66	0.04	Vergin et al., 2013
1994-02-18	65-0	20.358	36.612	222.010	0	0	0	0	0.95	5.49	0.14	Vergin et al., 2013
1997-09-12	108-0	27.037	36.703	199.560	0	0	0.01	0	0.94	3.66	0.03	Vergin et al., 2013
1997-10-07	109-0	26.600	36.406	201.500	0	0	0	0	0.35	4.43	0.05	Vergin et al., 2013
1997-11-12	110-0	24.158	36.599	209.100	0	0	0	0	0.46	4.84	0.08	Vergin et al., 2013
1997-12-09	111-0	22.060	36.530	212.850	0	0	0.02	0	0.82	5.14	0.21	Vergin et al., 2013
1998-01-13	112-0	21.890	36.760	216.140	0	0	0	0	0.62	5.27	0.08	Vergin et al., 2013
1998-02-12	113-0	20.380	36.682	219.550	0.06	0.02	0.04	0	0.63	4.55	0.26	Vergin et al., 2013
1998-03-12	114-0	19.590	36.622	225.700	0	0	0.02	0	0.65	3.56	0.27	Vergin et al., 2013
1998-04-07	115-0	19.990	36.653	223.840	0	0	0.01	0	0.92	5.19	0.15	Vergin et al., 2013
1998-05-05	116-0	20.960	36.668	222.030	0	0	0.01	0	0.77	3.99	0.09	Vergin et al., 2013
1998-06-01	117-0	23.080	36.450	216.350	0.06	0.05	0.01	0	-	5.78	0.08	Vergin et al., 2013
1998-07-07	118-0	26.730	36.310	204.060	0	0	0	0	0.41	5.29	0.07	Vergin et al., 2013
1998-09-10	120-0	28.140	36.190	197.890	0	0	0	0	0.35	5.86	0.08	Vergin et al., 2013
1998-12-08	123-0	23.090	36.560	213.050	0	0	0	0	0.55	4.18	0.07	Vergin et al., 2013

1999-01-15	124-0	21.910	36.500	217.440	0	0	0	0	1.03	5.76	0.08	Vergin et al., 2013
1999-02-12	125-0	21.430	36.650	216.720	0	0	0	0	0.69	5.74	0.18	Vergin et al., 2013
1999-03-23	126-0	19.480	36.300	226.300	0.06	0.06	0	0	0	2.76	0.41	Vergin et al., 2013
1999-04-07	127-0	19.710	36.650	223.230	0.1	0.04	0.06	0	0	-	0.35	Vergin et al., 2013
1999-05-04	128-0	21.483	36.580	220.500	0	0	0	0	0	-	0.07	Vergin et al., 2013
1999-06-01	129-0	22.910	36.580	215.820	-	-	0	-	0	5.34	0.04	Vergin et al., 2013
1999-07-06	130-0	27.150	36.780	202.150	0	0	0.01	0	0.72	3.22	0.04	Vergin et al., 2013
1999-08-03	131-0	27.400	36.610	200.580	0	0	0	0	0.38	6.05	0.06	Vergin et al., 2013
1999-09-15	132-0	27.790	36.360	198.370	0.07	0.07	0	0	0.44	5.46	0.04	Vergin et al., 2013
1999-10-12	133-0	26.260	36.500	216.010	0	0	0	0	0	5.05	0.03	Vergin et al., 2013
1999-11-09	134-0	24.490	36.560	206.900	0	0	0	0	0.4	4.81	0.11	Vergin et al., 2013
1999-12-10	135-0	22.810	36.810	209.980	0	0	0.01	0	0.41	5	0.14	Vergin et al., 2013
2000-01-29	136-0	20.520	36.750	215.460	0	0	0	0	0.46	4.56	0.22	Vergin et al., 2013
2000-02-15	137-0	19.960	36.670	220.670	0	0	0	0	0.35	6.97	0.09	Vergin et al., 2013
2000-03-14	138-0	19.360	36.650	226.050	0.11	0.11	0	0	0.72	6.51	0.16	Vergin et al., 2013
2000-04-11	139-0	19.930	36.660	229.290	0	0	0	0	0.93	9.94	0.15	Vergin et al., 2013
2000-05-10	140-0	22.020	36.600	222.000	0	0	0	0	0.81	6.8	0.05	Vergin et al., 2013
2000-06-06	141-0	24.930	36.830	208.540	0	-	-	0	-	2.83	0.01	Vergin et al., 2013
2000-07-11	142-0	26.510	36.490	202.980	0	0	0	0	0.65	4.19	0.02	Vergin et al., 2013
2000-08-08	143-0	28.910	36.540	202.120	0	0	0	0	0	4.19	0.01	Vergin et al., 2013
2000-09-13	144-0	27.840	36.070	197.570	0	-	0.01	0	0.23	3.97	0.02	Vergin et al., 2013
2000-10-18	145-0	26.140	36.680	203.370	-	-	-	-	0	2.97	0.02	Vergin et al., 2013
2000-11-14	146-0	23.360	36.660	210.340	0	0	0	0	0.34	3.1	0.11	Vergin et al., 2013
2000-12-12	147-0	21.990	36.600	214.810	0.06	0.06	0	-	0.41	3.64	0.02	Vergin et al., 2013
2001-01-30	148-0	20.140	36.610	219.570	0.06	0.03	0.03	0	0.43	3.43	0.27	Vergin et al., 2013
2001-02-20	149-0	19.930	36.650	222.190	0	0	0.01	0	0.24	4.7	0.24	Vergin et al., 2013
2001-03-09	150-0	19.680	36.690	220.690	0.06	0.03	0.03	-	-	3.56	0.21	Vergin et al., 2013
2001-04-04	151-0	20.130	36.720	224.570	0	0	0.01	0	1.25	4.84	0.05	Vergin et al., 2013
2001-05-15	152-0	21.340	36.780	219.520	0.23	0.22	0.01	0	0.47	3.66	0.04	Vergin et al., 2013
2001-06-05	153-0	24.470	36.850	210.870	0	0	0	0	1.32	3.05	0.05	Vergin et al., 2013
2001-06-01	153-0	24.470	36.850	210.870	0	0	0	0	1.32	3.05	0.05	Vergin et al., 2013
2001-07-18	154-0	26.500	36.500	208.420	0	0	0	0	1.14	4.04	0.07	Vergin et al., 2013
2001-08-07	155-0	27.550	36.530	201.250	0	0	0	0	1.06	3.56	0.06	Vergin et al., 2013
2001-09-12	156-0	27.560	36.520	202.340	0	0	0	0	1.22	3.04	0.06	Vergin et al., 2013
2001-10-16	157-0	24.680	36.440	209.770	0	0	0	0	0.53	4.63	0.1	Vergin et al., 2013
2001-11-09	158-0	24.370	36.550	209.940	0	0	0	0	0.46	3.84	0.06	Vergin et al., 2013
2001-12-11	159-0	22.280	36.620	215.590	0	0	0	0.04	0.66	3.7	0.12	Vergin et al., 2013
2002-01-01	160-0	-	-	-	-	-	-	-	-	-	-	Vergin et al., 2013
2002-02-13	161-0	20.350	36.690	221.270	0	0	0.02	0.04	0.47	3.2	0.26	Vergin et al., 2013
2002-03-19	162-0	19.940	36.670	226.390	0	0	0.01	0	0.66	4.72	0.13	Vergin et al., 2013
2002-04-16	163-0	21.250	36.760	221.310	0	0	0	0.04	0.7	3.62	0.05	Vergin et al., 2013
2002-05-14	164-0	21.720	36.520	222.860	0	0	0	0	-	3.68	0.07	Vergin et al., 2013
2002-06-04	165-0	23.490	36.470	215.140	0	0	0	0	0.25	3.71	0.05	Vergin et al., 2013
2002-07-02	166-0	26.870	36.620	202.570	0	0	0	0	0.81	1.97	0.05	Vergin et al., 2013
2002-08-20	167-0	27.870	36.320	200.520	0	0	0	0	1.32	4.64	-	Vergin et al., 2013

2002-09-17	168-0	26.990	36.210	198.250	0	0	0	0	0	4.48	0.06	Vergin et al., 2013
2002-10-08	169-0	26.410	36.190	202.930	0	0	0	0.06	0.27	4.49	0.02	Vergin et al., 2013
2002-11-13	170-0	24.680	36.530	209.910	0	0	0.01	0	0.92	4.16	0.07	Vergin et al., 2013
2002-12-10	171-0	23.400	36.660	211.680	0	0	0	0	0.7	3.98	0.17	Vergin et al., 2013
2003-01-22	172-0	20.820	36.720	218.410	0	0	0.03	0	0.63	3.9	0.14	Vergin et al., 2013
2003-02-21	173-0	20.600	36.740	222.010	0	0	0	0	1.08	4.27	0.16	Vergin et al., 2013
2003-03-21	174-0	19.990	36.670	224.820	0	0	0.01	0	1.44	4.86	0.06	Vergin et al., 2013
2003-04-22	175-0	21.770	36.580	219.310	0	0	0	0	0.57	4.78	0.06	Vergin et al., 2013
2003-05-20	176-0	22.700	36.680	216.600	0	0	0	0	0.63	4.85	0.06	Vergin et al., 2013
2003-06-01	177-0	26.760	36.390	207.530	0	0	0	0	0.6	3.98	0.02	Vergin et al., 2013
2003-07-15	178-0	27.830	36.450	204.520	-	-	-	-	-	4.44	0.03	Vergin et al., 2013
2003-08-12	179-0	28.860	36.640	198.980	0	0	0	0	0.7	4.48	0.05	Vergin et al., 2013
2003-09-19	180-0	26.170	36.470	210.010	0	0	0	0	0.91	4.77	0.09	Vergin et al., 2013
2003-10-07	181-0	26.350	36.392	201.800	0	0	0	0.04	0.94	3.93	0.08	Vergin et al., 2013
2003-12-02	183-0	24.210	36.800	208.850	0	0	0	0	0.99	4.3	0.14	Vergin et al., 2013
2004-01-27	184-0	20.300	36.693	219.800	0.11	0.11	0	0	0.9	4.38	0.39	Vergin et al., 2013
2016-03-08	10321-1_S39	21.151	36.671	314.200	0	0	0.02	0	0	7.7	0.11	BATS this study
2016-03-25	10322-1_S51	20.899	36.624	-	-	-	-	-	-	-	0.1	BATS this study
2016-04-04	20322-1_S63	21.533	36.664	218.200	-	-	-	-	-	-	0.08	BATS this study
2016-04-15	SC1_S91	21.350	36.701	221.100	0	0	0	0	0.35	6.8	0.06	BATS this study
2016-05-03	SC23_S113	21.789	36.647	220.900	0	0	0.01	0.05	0	6.4	0.08	BATS this study
2016-07-10	C7_N1_S112	26.674	36.336	204.786	0.04	-	-	0.02	-	7	-	BATS this study
2016-08-17	10327-1_S89	29.384	36.508	196.600	0	-	-	0	0.6	3.3	0.03	BATS this study
2016-09-22	10328-1_S101	28.776	36.706	195.400	0	0	0	0	0.68	3	0.04	BATS this study
2016-10-20	BS-1_S62	26.158	36.815	206.000	0	0	0.02	0	0	4.2	0.07	BATS this study
2016-11-21	BS-13_S74	22.952	36.629	-	0	-	-	0	0	3.8	0.1	BATS this study
2016-12-15	BS-25_S86	21.765	36.557	216.700	0	0	0.02	0	0	4.5	0.11	BATS this study
2017-01-07	BS-37_S98	21.654	36.628	219.366	0	0	0.01	0	0.91	6.62	0.11	BATS this study
2017-02-19	BS-49_S110	21.107	36.777	218.267	-	-	-	-	-	5.15	0.16	BATS this study
2017-04-10	BS-61_S122	20.454	36.740	223.870	0.19	0	0.19	0.07	0.97	4.38	0.17	BATS this study
2017-04-18	BS-73_S134	21.277	36.845	220.859	-	-	-	-	-	4.23	0.05	BATS this study
2017-05-10	BS-81_S142	23.187	36.834	214.562	0	0	0	0	0.51	4.34	0.03	BATS this study
2017-05-30	BS93_S1	24.090	36.742	215.308	-	-	-	-	-	4.14	0.05	BATS this study
2017-06-14	BS101_S9	25.936	36.738	209.371	-	-	-	-	-	6.87	0.05	BATS this study
2017-07-19	BS113_S21	28.230	36.612	200.337	0	0	0	0	0.39	6.2	0.05	BATS this study
2017-08-17	BS124_S32	29.054	36.832	196.921	0	0	0.22	0.04	0.85	6.01	0.04	BATS this study
2017-09-14	BS135_S43	28.391	36.522	196.308	0	0	0.01	0	0.97	6.48	0.05	BATS this study
2017-10-17	BS147_S55	25.944	36.518	205.366	0	0	0.01	0	1.03	6.3	0.06	BATS this study
2017-11-18	BS159_S67	24.471	36.538	206.241	0	0	0.01	0	1.1	4.63	0.05	BATS this study
2017-12-14	BS171_S79	23.132	36.664	210.645	0	0	0.01	0	0.86	4.14	0.12	BATS this study
2018-01-17	BS_183_S21	21.787	36.681	216.803	-	-	-	0	0.88	4.44	0.07	BATS this study
2018-02-12	BS_195_S33	21.065	36.793	221.450	0	0	0	0	0.79	6.35	0.09	BATS this study
2018-02-26	BS_207_S45	21.333	36.805	221.305	0	0	0.01	0	0.81	-	0.02	BATS this study
2018-03-25	BS_215_S53	20.186	36.781	221.793	-	-	-	-	-	5.3	0.16	BATS this study
2018-04-16	BS_227_S65	20.880	36.782	225.881	-	-	-	-	-	1.85	0.05	BATS this study

2018-04-25	BS_239_S77	20.993	36.796	228.764	0	0	0.01	0	0.83	6.75	0.09	BATS this study
2018-05-25	BS_247_S85	24.414	36.690	213.043	0	0	0	0	0.79	5.17	0.03	BATS this study
2018-06-14	BS_259_S97	25.415	36.550	207.924	0	0	0	0	0.9	3.81	0.04	BATS this study
2018-07-21	BS271_S109	26.992	36.534	201.715	0	0	0	0	0.87	3.89	0.02	BATS this study
2018-08-14	BS283_S121	28.358	36.591	197.078	0	0	0	0	1.01	3.77	0.01	BATS this study
2018-09-12	BS295_S133	28.721	36.665	200.157	0	0	0	0	0.88	4.09	0.03	BATS this study
2018-10-21	BS307_S145	25.713	36.960	204.511	0	0	0	0	0.81	4.77	0.04	BATS this study
2018-11-07	BS319_S157	24.514	36.608	209.879	0	0	0	0	0.94	6.8	0.06	BATS this study
2018-12-15	BS330_S167	22.336	36.793	215.435	0	0	0	0	0.81	-	0.07	BATS this study

Wester English Channel Time-series

Date (YYYY-MM-DD)	Sample	Temperature (°C)	Salinity (p.s.u.)	O2 (µmol/kg)	NO2+NO3 (µmol/kg)	NO2 (µmol/kg)	NH4 (µmol/kg)	PO4 (µmol/kg)	SiO2 (µmol/kg)	Bacteria (cells*10^8/kg)	Chl.a (µg/L)	Reference
2012-02-06	KT16S193	9.486	35.290	357.648	6.910	0.255	-	0.440	3.325	-	0.310	WEC this study
2012-02-13	KT16S194	9.210	35.110	272.024	7.610	0.305	0.205	0.455	3.910	-	0.400	WEC this study
2012-02-20	KT16S195	9.129	35.166	273.651	7.603	0.310	0.130	0.540	3.868	-	0.610	WEC this study
2012-02-27	KT16S196	9.366	35.117	275.953	8.100	0.373	-	0.525	3.563	-	0.680	WEC this study
2012-03-12	KT16S197	9.571	35.255	274.137	6.103	0.395	0.373	0.450	3.300	-	0.860	WEC this study
2012-03-19	KT16S198	9.559	35.162	282.236	5.795	0.330	0.168	0.378	3.133	-	1.770	WEC this study
2012-03-26	KT16S199	9.762	35.302	278.651	5.345	0.278	0.498	0.350	-	-	1.770	WEC this study
2012-04-02	KT16S200	10.102	35.327	294.946	2.695	0.125	0.270	0.240	1.175	-	4.350	WEC this study
2012-04-16	KT16S201	10.144	35.332	301.453	0.295	0.058	0.230	0.125	0.215	-	3.610	WEC this study
2012-04-23	KT16S202	10.337	35.257	281.601	0.105	0.060	0.200	0.085	0.268	-	2.730	WEC this study
2012-04-30	KT16S203	10.314	35.182	273.332	0.320	0.065	0.245	0.133	1.393	-	2.120	WEC this study
2012-05-07	KT16S204	10.902	35.259	272.072	0.413	0.050	0.798	0.113	1.583	-	1.130	WEC this study
2012-05-21	KT16S205	11.390	35.124	317.496	0.078	0.013	-	0.040	0.325	-	2.450	WEC this study
2012-05-28	KT16S206	-	-	-	-	-	-	-	-	-	-	WEC this study
2012-06-11	KT16S207	12.794	35.076	272.388	0.095	0.020	0.395	0.075	0.348	-	3.130	WEC this study
2012-06-25	KT16S208	13.941	34.638	264.453	-	-	-	-	-	-	2.450	WEC this study
2012-07-02	KT16S209	14.074	35.085	251.719	0.085	0.010	0.168	0.030	0.185	-	1.390	WEC this study
2012-07-09	KT16S210	15.080	34.534	253.191	2.670	0.070	0.338	0.080	1.360	-	1.480	WEC this study
2012-07-23	KT16S211	15.973	35.069	263.852	0.040	0.000	0.000	0.023	0.105	-	0.630	WEC this study
2012-07-30	KT16S212	15.053	35.089	267.397	0.000	0.000	0.635	0.038	0.525	-	2.050	WEC this study
2012-08-06	KT16S213	15.419	35.171	235.778	0.115	0.000	0.615	0.060	0.395	-	1.610	WEC this study
2012-08-13	KT16S214	17.382	35.201	243.745	0.070	0.015	0.678	0.045	0.368	-	0.570	WEC this study
2012-08-20	KT16S215	17.806	34.961	231.600	0.950	0.095	1.210	0.160	1.973	-	1.100	WEC this study
2012-09-17	KT16S216	16.117	35.150	247.453	0.055	0.020	0.655	0.083	0.303	-	1.340	WEC this study
2012-10-08	KT16S217	14.783	35.133	241.796	1.900	0.523	0.310	0.280	1.950	-	2.000	WEC this study
2012-10-15	KT16S218	-	-	-	5.835	1.110	0.175	0.355	3.580	-	1.080	WEC this study
2012-10-29	KT16S219	14.035	35.083	235.807	4.153	1.025	0.645	0.335	3.760	-	0.580	WEC this study
2012-11-12	KT16S220	13.002	35.046	243.352	4.708	0.513	0.090	0.370	4.038	-	0.660	WEC this study
2012-11-26	KT16S221	11.788	34.257	254.687	8.885	0.210	0.210	0.478	5.915	-	0.470	WEC this study
2012-12-03	KT16S222	-	-	-	6.640	0.145	0.080	0.440	4.775	-	0.600	WEC this study
2012-12-10	KT16S223	11.460	34.766	256.580	6.585	0.155	0.340	0.430	5.510	-	0.620	WEC this study
2012-12-17	KT16S224	-	-	-	7.048	0.170	-	0.470	5.485	-	0.550	WEC this study
2013-01-07	KT16S225	10.625	34.507	272.520	8.435	0.095	-	0.535	5.265	-	0.650	WEC this study

2013-01-11	KT16S255	-	-	-	-	-	-	-	-	-	-	WEC this study
2013-01-21	KT16S226	-	-	-	7.015	0.070	0.465	0.498	4.940	-	0.330	WEC this study
2013-02-04	KT16S227	-	-	-	-	-	-	-	-	-	0.360	WEC this study
2013-02-11	KT16S228	9.099	34.662	273.129	9.528	0.210	0.080	0.520	5.778	-	0.500	WEC this study
2013-02-18	KT16S229	-	-	-	7.928	0.180	0.140	0.565	4.913	-	0.410	WEC this study
2013-02-25	KT16S230	8.352	34.830	281.505	8.198	0.190	0.100	0.510	5.843	-	0.410	WEC this study
2013-03-11	KT16S231	8.217	35.156	283.282	6.635	0.180	0.280	0.425	4.100	-	0.660	WEC this study
2013-03-18	KT16S232	7.985	34.760	289.499	7.915	0.190	0.338	0.500	5.123	-	1.090	WEC this study
2013-04-01	KT16S233	7.629	35.106	286.052	6.988	0.230	-	0.480	3.845	-	0.520	WEC this study
2013-04-29	KT16S234	8.220	35.035	289.391	6.845	0.190	0.530	0.430	3.558	-	3.340	WEC this study
2013-05-06	KT16S235	-	-	-	2.967	0.135	0.363	0.180	2.063	-	3.150	WEC this study
2013-05-13	KT16S236	-	-	-	2.023	0.090	0.713	0.150	0.363	-	2.070	WEC this study
2013-05-20	KT16S237	10.476	34.920	317.571	0.220	0.030	0.293	0.063	0.298	-	2.650	WEC this study
2013-05-27	KT16S238	10.365	35.191	316.298	0.055	0.000	0.333	0.055	0.670	-	0.280	WEC this study
2013-06-03	KT16S239	11.573	35.169	313.445	0.015	0.000	0.300	0.120	0.850	-	0.100	WEC this study
2013-06-10	KT16S240	-	-	-	-	-	-	-	-	-	0.510	WEC this study
2013-06-17	KT16S241	-	-	-	0.060	0.010	0.123	0.055	0.800	-	0.740	WEC this study
2013-06-24	KT16S242	12.311	35.029	272.140	0.000	0.000	0.258	0.045	-	-	1.260	WEC this study
2013-07-01	KT16S243	13.139	35.165	271.387	0.073	0.025	0.047	0.040	1.330	-	0.460	WEC this study
2013-07-08	KT16S244	15.741	35.166	279.836	0.027	0.023	0.020	0.010	0.203	-	0.440	WEC this study
2013-07-15	KT16S245	17.240	35.249	270.267	0.037	0.010	0.000	0.010	0.188	-	0.790	WEC this study
2013-09-02	KT16S246	16.392	35.130	197.876	0.013	0.000	-	0.020	1.420	-	0.960	WEC this study
2013-09-23	KT16S247	15.134	35.164	242.623	1.813	0.500	0.230	0.163	1.803	-	-	WEC this study
2013-09-30	KT16S248	-	-	-	2.053	0.930	0.350	0.210	2.473	-	0.980	WEC this study
2013-10-07	KT16S249	16.226	35.132	251.965	1.198	0.555	0.130	0.195	3.150	-	1.750	WEC this study
2013-11-04	KT16S250	13.930	34.968	233.630	6.413	0.605	0.130	0.393	3.918	-	0.500	WEC this study
2013-11-11	KT16S251	-	-	-	6.640	0.505	0.105	0.348	3.925	-	0.780	WEC this study
2013-11-18	KT16S252	13.103	34.783	245.186	7.233	0.285	0.180	0.385	4.335	-	0.510	WEC this study
2013-12-09	KT16S253	-	-	-	6.853	0.270	0.098	0.415	3.150	-	0.330	WEC this study
2013-12-16	KT16S254	11.636	35.066	259.403	7.028	0.150	0.233	0.438	4.288	-	0.510	WEC this study
2014-01-20	KT16S256	9.531	33.459	269.022	16.230	0.260	1.033	0.710	7.953	-	0.320	WEC this study
2014-01-27	KT16S257	9.802	34.840	265.693	9.983	0.190	0.570	0.590	5.253	-	0.310	WEC this study
2014-02-10	KT16S258	-	-	-	-	-	-	-	-	-	-	WEC this study
2014-03-03	KT16S259	8.895	34.869	279.671	9.850	0.130	0.505	0.658	5.728	-	0.350	WEC this study
2014-03-24	KT16S260	9.397	35.067	277.514	8.730	0.230	0.423	0.625	5.350	-	0.410	WEC this study
2014-03-31	KT16S261	9.484	35.093	275.929	8.990	0.320	0.430	0.610	4.958	-	0.410	WEC this study
2014-04-07	KT16S262	10.209	34.842	246.134	9.020	0.340	0.373	0.495	4.540	-	0.500	WEC this study
2014-04-14	KT16S263	10.423	34.625	308.103	4.403	0.310	0.313	0.205	0.988	-	3.840	WEC this study
2014-05-12	KT16S264	10.868	35.277	275.149	3.285	0.135	1.085	0.395	1.605	-	1.870	WEC this study
2014-05-19	KT16S265	12.405	35.151	224.350	0.015	0.000	0.400	0.100	0.463	-	0.420	WEC this study
2014-06-09	KT16S266	14.068	35.178	-	0.020	0.008	0.533	0.040	0.080	-	0.410	WEC this study
2014-06-16	KT16S267	15.620	35.214	-	0.025	0.000	0.315	0.028	0.095	-	0.490	WEC this study
2014-06-23	KT16S268	17.200	35.234	-	0.095	0.010	0.177	0.105	0.143	-	0.370	WEC this study
2014-06-30	KT16S269	16.093	35.099	254.968	0.025	0.005	0.198	0.018	0.343	-	0.920	WEC this study
2014-07-07	KT16S270	17.071	35.097	249.579	0.035	0.015	0.168	0.020	0.060	-	0.630	WEC this study

2014-07-14	KT16S271	15.938	35.090	245.958	0.000	0.000	0.490	0.060	0.703	-	0.520	WEC this study
2014-07-21	KT16S272	17.109	35.095	270.438	0.015	0.010	0.253	0.040	0.188	-	0.450	WEC this study
2014-08-18	KT16S273	15.188	35.079	246.912	1.145	0.255	0.265	0.125	2.205	-	1.020	WEC this study
2014-08-25	KT16S274	15.689	35.093	245.194	0.400	0.085	0.380	0.055	1.803	-	0.580	WEC this study
2014-09-08	KT16S275	16.762	34.545	258.082	2.020	0.015	0.020	0.015	-	-	0.350	WEC this study
2014-09-15	KT16S276	-	-	-	1.430	0.550	0.433	0.135	-	-	0.490	WEC this study
2014-09-22	KT16S277	17.024	35.133	251.638	0.158	0.035	0.150	0.055	1.883	-	0.410	WEC this study
2014-09-29	KT16S278	17.242	35.160	238.548	0.580	0.140	0.273	0.118	2.335	-	0.510	WEC this study
2014-10-13	KT16S279	16.077	35.163	225.881	2.518	0.980	0.545	0.280	3.145	-	0.290	WEC this study
2014-10-20	KT16S280	-	-	-	-	-	-	-	-	-	0.240	WEC this study
2014-10-24	KT16S281	-	-	-	-	-	-	-	-	-	-	WEC this study
2014-11-03	KT16S282	14.469	34.896	235.777	6.278	0.730	0.253	0.428	4.717	-	0.350	WEC this study
2014-11-17	KT16S283	13.392	34.059	244.412	9.133	0.475	0.345	0.435	5.580	-	0.230	WEC this study
2014-11-24	KT16S284	13.600	35.223	240.879	4.850	0.317	0.143	0.373	2.763	-	0.230	WEC this study
2014-12-01	KT16S285	13.186	35.158	242.025	5.490	0.143	0.093	0.410	2.940	-	0.150	WEC this study
2014-12-08	KT16S286	12.478	35.120	247.546	6.143	0.190	0.085	0.398	3.100	-	0.170	WEC this study
2014-12-15	KT16S287	-	-	-	6.063	0.105	0.128	0.418	3.497	-	0.110	WEC this study
2015-01-05	KT16S288	11.233	35.143	260.775	6.965	0.095	0.100	0.470	4.153	-	0.130	WEC this study
2015-01-19	KT16S289	10.972	35.262	259.138	6.795	0.130	0.140	0.465	3.100	-	0.170	WEC this study
2015-02-02	KT16S290	10.015	35.236	262.651	7.278	0.165	0.103	0.445	3.390	-	0.200	WEC this study
2015-02-09	KT16S291	9.339	35.194	267.644	7.403	0.250	0.225	0.440	3.420	-	0.270	WEC this study
2015-02-16	KT16S292	9.237	35.208	270.166	7.515	0.190	0.238	0.460	3.420	-	0.250	WEC this study
2015-03-09	KT16S293	9.225	35.146	285.271	5.143	0.198	-	0.323	1.915	-	1.650	WEC this study
2015-03-16	KT16S294	-	-	-	4.810	0.180	0.343	0.333	1.358	-	2.030	WEC this study
2015-03-23	KT16S295	9.137	35.249	284.623	3.868	0.140	0.285	0.283	0.908	-	1.440	WEC this study
2015-04-06	KT16S296	9.487	35.239	282.466	2.910	0.120	0.710	0.248	1.178	-	1.790	WEC this study
2015-04-20	KT16S297	10.182	35.219	282.821	1.318	0.070	0.905	0.158	1.510	-	0.740	WEC this study
2015-04-27	KT16S298	10.411	35.298	290.690	0.015	0.000	0.173	0.060	1.013	-	1.180	WEC this study
2015-05-04	KT16S299	10.877	35.189	268.598	0.275	0.015	-	0.095	1.213	-	2.040	WEC this study
2015-05-11	KT16S300	11.657	35.092	268.719	0.615	0.035	-	0.130	1.480	-	2.260	WEC this study
2015-05-18	KT16S301	11.214	35.232	268.879	0.160	0.015	-	0.105	0.730	-	3.140	WEC this study
2015-05-27	KT16S302	-	-	-	-	-	-	-	-	-	-	WEC this study
2015-06-01	KT16S303	11.497	35.305	260.190	0.070	0.005	-	0.120	0.835	-	2.420	WEC this study
2015-06-08	KT16S304	12.600	35.202	272.638	0.000	0.000	-	0.075	0.330	-	1.390	WEC this study
2015-06-15	KT16S305	13.306	35.283	267.371	0.000	0.000	0.193	0.035	0.295	-	0.590	WEC this study
2015-06-22	KT16S306	13.634	35.266	267.581	0.000	0.000	0.218	0.065	0.225	-	0.900	WEC this study
2015-06-29	KT16S307	15.582	35.283	258.867	0.010	0.005	-	0.045	-	-	0.430	WEC this study
2015-07-06	KT16S308	15.728	35.296	257.009	0.010	0.010	-	0.030	0.165	-	0.860	WEC this study
2015-07-13	KT16S309	15.170	35.236	258.235	0.007	0.000	0.070	0.030	0.558	-	1.520	WEC this study
2015-07-20	KT16S310	16.173	35.250	269.224	0.000	0.000	0.000	0.000	0.625	-	0.910	WEC this study
2015-07-28	KT16S311	-	-	-	-	-	-	-	-	-	-	WEC this study
2015-08-03	KT16S312	15.664	35.221	259.787	0.000	0.000	0.410	0.000	0.265	-	1.530	WEC this study
2015-08-10	KT16S313	15.823	35.171	264.727	0.000	0.000	0.308	0.000	0.260	-	2.290	WEC this study
2015-08-17	KT16S314	16.702	35.247	267.294	0.000	0.000	0.148	0.000	0.488	-	-	WEC this study
2015-08-24	KT16S315	16.584	35.162	240.779	0.000	0.000	0.300	0.050	0.570	-	0.980	WEC this study

2015-08-31	KT16S316	15.847	35.101	234.150	0.833	0.140	0.617	0.133	2.028	-	1.090	WEC this study
2015-09-07	KT16S317	15.378	35.286	234.963	1.140	0.500	0.105	0.118	1.515	-	2.040	WEC this study
2015-09-14	KT16S318	-	-	-	0.115	0.045	0.000	0.078	1.360	-	2.510	WEC this study
2015-09-21	KT16S319	-	-	-	0.450	0.150	0.145	0.110	1.850	-	1.680	WEC this study
2015-09-28	KT16S320	15.623	35.219	236.715	0.093	0.060	0.037	0.095	2.290	-	2.020	WEC this study
2015-10-05	KT16S321	15.430	35.224	234.349	0.240	0.160	0.153	0.093	2.153	-	1.010	WEC this study
2015-10-12	KT16S322	15.200	35.228	227.175	1.225	0.735	0.308	0.165	2.510	-	0.720	WEC this study
2015-10-19	KT16S323	14.955	35.306	231.971	1.243	0.915	0.127	0.155	1.953	-	1.490	WEC this study
2015-10-26	KT16S324	-	-	-	1.538	0.855	0.125	0.190	2.280	-	1.180	WEC this study
2015-11-02	KT16S325	14.617	35.302	233.253	1.990	0.530	0.123	0.233	2.193	-	0.910	WEC this study
2015-11-16	KT16S326	13.974	35.214	238.783	4.133	0.050	0.020	0.340	3.255	-	0.570	WEC this study
2015-11-23	KT16S327	13.512	35.195	238.740	4.453	0.105	0.140	0.348	3.168	-	0.520	WEC this study
2015-12-07	KT16S328	12.597	35.255	251.008	5.758	0.050	0.350	0.453	3.257	-	0.270	WEC this study
2015-12-14	KT16S329	12.300	35.232	252.096	6.543	0.115	0.108	0.478	3.410	-	0.340	WEC this study
2016-01-04	KT16S330	11.668	35.060	252.222	6.048	0.095	0.175	0.520	4.010	-	0.320	WEC this study
2016-01-11	KT16S331	11.124	35.064	253.197	6.098	0.075	0.063	0.485	3.888	-	0.400	WEC this study
2016-01-18	KT16S332	10.757	35.027	254.297	7.065	0.080	0.088	0.493	3.913	-	0.370	WEC this study
2016-01-25	KT16S333	10.255	34.410	264.533	11.700	0.175	0.457	0.593	5.585	-	0.500	WEC this study
2016-02-01	KT16S334	-	-	-	8.250	0.070	0.130	0.528	4.425	-	0.500	WEC this study
2016-02-08	KT16S335	10.303	35.113	259.368	7.378	0.090	0.150	0.543	3.685	-	-	WEC this study
2016-02-15	KT16S336	10.204	35.107	259.207	7.095	0.090	0.000	0.515	3.455	-	0.430	WEC this study
2016-02-22	KT16S337	9.879	34.926	265.528	8.048	0.165	0.163	0.525	3.848	-	0.570	WEC this study
2016-02-29	KT16S338	9.785	35.069	265.830	7.353	0.145	0.133	0.500	3.570	-	0.540	WEC this study
2016-03-07	KT16S339	9.451	35.013	266.768	7.473	0.235	0.135	0.473	3.663	-	0.690	WEC this study
2016-03-14	KT16S340	9.346	34.937	277.219	6.878	0.255	0.150	0.475	3.148	-	0.640	WEC this study
2016-03-21	KT16S341	9.536	35.097	269.154	7.127	0.220	0.060	0.448	3.573	-	0.950	WEC this study
2016-03-28	KT16S342	9.460	34.970	269.982	5.705	0.200	0.355	0.430	2.910	-	1.100	WEC this study
2016-04-04	KT16S343	9.593	35.015	270.780	4.650	0.233	0.440	0.410	1.988	-	0.840	WEC this study
2016-04-11	KT16S344	9.766	35.047	267.806	5.958	0.285	0.390	0.410	2.720	-	0.610	WEC this study
2016-04-18	KT16S345	9.999	34.928	270.794	5.170	0.180	0.165	0.333	2.580	-	1.460	WEC this study
2016-04-25	KT16S346	10.164	34.967	276.345	5.530	0.200	0.277	0.388	2.163	-	1.330	WEC this study
2016-05-02	KT16S347	10.242	35.037	288.808	1.985	0.140	0.410	0.195	0.990	-	1.360	WEC this study
2016-05-09	KT16S348	10.635	35.166	300.819	0.180	0.025	0.190	0.103	0.655	-	2.310	WEC this study
2016-05-16	KT16S349	11.804	35.219	305.560	0.000	0.000	0.188	0.075	0.715	-	0.400	WEC this study
2016-05-23	KT16S350	12.061	35.057	276.109	0.000	0.000	0.107	0.065	1.187	-	0.300	WEC this study
2016-05-30	KT16S351	12.783	35.165	267.811	0.010	0.005	0.263	0.045	0.935	-	0.190	WEC this study
2016-06-09	KT16S352	-	-	-	-	-	-	-	-	-	-	WEC this study
2016-06-20	KT16S353	14.058	34.943	253.370	0.015	0.000	0.050	0.028	0.238	-	0.520	WEC this study
2016-06-27	KT16S354	13.810	35.166	256.653	0.013	0.000	0.087	0.015	0.563	-	0.870	WEC this study
2016-07-04	KT16S355	14.458	35.016	258.718	0.020	0.000	0.127	0.000	0.538	-	0.880	WEC this study
2016-07-11	KT16S356	14.652	35.158	254.262	0.000	0.000	0.090	0.015	1.260	-	1.130	WEC this study
2016-07-18	KT16S357	16.244	35.222	253.382	0.000	0.000	0.190	0.025	1.200	-	0.480	WEC this study
2016-07-25	KT16S358	16.760	35.253	279.705	0.000	0.000	0.195	0.000	1.043	-	1.530	WEC this study
2016-08-01	KT16S359	16.125	35.235	255.200	0.020	0.000	0.093	0.050	1.108	-	1.280	WEC this study
2016-08-08	KT16S360	15.544	35.139	260.108	0.000	0.000	0.018	0.010	1.655	-	0.860	WEC this study

2016-08-15	KT16S361	16.232	35.213	265.939	0.020	0.000	0.020	0.000	1.550	-	0.840	WEC this study
2016-08-22	KT16S362	16.651	35.197	245.543	0.005	0.000	0.218	0.040	1.890	-	3.840	WEC this study
2016-08-29	KT16S363	16.891	35.273	234.857	0.030	0.015	0.225	-	1.207	-	1.050	WEC this study
2016-09-05	KT16S364	16.483	35.178	228.534	0.303	0.160	0.457	0.095	1.438	-	1.510	WEC this study
2016-09-12	KT16S365	17.079	35.116	234.464	0.030	0.020	0.400	0.040	0.883	-	1.400	WEC this study
2016-09-19	KT16S366	16.253	35.101	177.332	0.655	0.190	0.133	0.103	1.373	-	2.500	WEC this study
2016-09-26	KT16S367	16.272	35.152	229.280	0.790	0.265	0.318	0.145	1.465	-	1.530	WEC this study
2016-10-10	KT16S368	15.803	35.283	228.833	1.345	0.520	0.108	0.150	1.388	-	1.590	WEC this study
2016-10-17	KT16S369	15.285	35.281	225.997	1.565	0.665	0.388	0.185	1.648	-	-	WEC this study
2016-10-24	KT16S370	-	-	-	1.095	0.820	0.353	0.210	1.455	-	0.940	WEC this study
2016-10-31	KT16S371	15.240	35.272	228.443	1.488	1.060	0.258	0.235	1.720	-	0.610	WEC this study
2016-11-07	KT16S372	14.771	35.306	229.158	1.995	1.160	0.148	0.250	1.820	-	0.950	WEC this study
2016-11-14	KT16S373	14.136	35.277	229.580	2.650	1.317	0.140	0.268	2.243	-	0.800	WEC this study
2016-11-21	KT16S374	12.517	34.469	239.213	-	-	-	-	-	-	1.090	WEC this study
2016-11-28	KT16S375	12.877	35.172	233.036	3.905	0.540	0.127	0.323	2.695	-	0.680	WEC this study
2016-12-05	KT16S376	12.128	35.077	240.751	4.640	1.385	0.018	0.488	4.673	-	0.630	WEC this study
2016-12-12	KT16S377	12.276	35.155	241.253	4.175	0.075	0.100	0.350	3.630	-	0.510	WEC this study
2016-12-19	KT16S378	12.300	35.097	199.094	4.078	0.075	0.123	0.380	3.440	-	0.630	WEC this study
2017-01-02	KT16S379	11.628	35.237	244.892	-	-	-	-	-	-	0.550	WEC this study
2017-01-09	KT16S380	10.924	35.089	247.747	6.103	0.228	0.155	0.428	3.633	-	0.510	WEC this study
2017-01-16	KT16S381	10.670	35.037	247.367	5.983	0.270	0.100	0.428	3.838	-	0.620	WEC this study
2017-01-23	KT16S382	10.380	35.171	251.196	5.543	0.178	0.000	0.413	-	-	0.800	WEC this study
2017-01-30	KT16S383	10.165	35.202	254.132	5.433	0.190	0.083	0.420	3.388	-	0.670	WEC this study
2017-02-06	KT16S384	9.622	34.744	256.172	7.735	0.323	0.313	0.490	4.660	-	0.670	WEC this study
2017-02-13	KT16S385	-	-	-	5.867	0.180	0.095	0.455	3.305	-	1.320	WEC this study
2017-02-20	KT16S386	9.756	34.861	261.753	8.048	0.265	0.178	0.448	3.708	-	0.810	WEC this study
2017-02-27	KT16S387	9.706	35.199	255.756	5.658	0.120	0.000	0.455	3.140	-	1.190	WEC this study
2017-03-06	KT16S388	9.675	35.174	256.202	5.653	0.165	-	0.425	2.750	-	0.800	WEC this study
2017-03-13	KT16S389	9.942	34.780	261.974	7.593	0.258	-	0.435	3.613	-	0.460	WEC this study
2017-03-20	KT16S390	10.158	35.149	217.058	5.695	0.280	0.163	0.410	2.650	-	0.490	WEC this study
2017-03-27	KT16S391	-	-	-	5.783	0.340	0.145	0.408	2.863	-	0.860	WEC this study
2017-04-03	KT16S392	10.717	35.125	266.164	5.123	0.285	0.125	0.355	2.543	-	1.930	WEC this study
2017-04-10	KT16S393	10.922	35.015	277.635	3.888	0.190	0.093	0.238	2.460	-	2.990	WEC this study
2017-04-17	KT16S394	-	-	-	2.427	0.190	0.350	0.253	1.763	-	1.330	WEC this study
2017-04-24	KT16S395	11.702	35.163	290.605	0.608	0.055	0.355	0.145	0.988	-	2.100	WEC this study
2017-05-01	KT16S396	10.999	35.230	264.882	0.883	0.090	0.810	0.225	0.520	-	1.000	WEC this study
2017-05-08	KT16S397	11.514	35.277	278.736	0.187	0.040	0.170	-	0.273	-	0.800	WEC this study
2017-05-15	KT16S398	12.336	34.981	259.053	0.000	0.000	0.000	0.103	0.233	-	0.760	WEC this study
2017-05-22	KT16S399	12.971	35.050	261.086	0.000	0.000	0.000	0.050	0.158	-	1.750	WEC this study
2017-05-29	KT16S400	13.730	35.131	266.637	0.000	0.000	0.020	0.015	0.097	-	2.680	WEC this study
2017-06-12	KT16S401	13.438	35.060	250.878	0.028	0.000	0.183	0.070	0.400	-	0.650	WEC this study
2017-06-19	KT16S402	16.290	35.207	253.371	0.000	0.000	0.148	0.000	0.133	-	0.890	WEC this study
2017-06-26	KT16S403	15.288	35.120	197.268	0.000	0.000	0.133	0.030	0.340	-	0.910	WEC this study
2017-07-03	KT16S404	14.962	35.263	252.908	0.000	0.000	-	0.035	0.145	-	0.390	WEC this study
2017-07-17	KT16S405	16.141	35.070	210.064	0.040	0.000	-	0.040	0.433	-	0.380	WEC this study

2017-07-24	KT16S406	16.642	35.110	231.812	0.190	0.035	-	0.068	1.127	-	1.080	WEC this study
2017-07-31	KT16S407	-	-	-	0.015	0.005	-	0.048	1.250	-	-	WEC this study
2017-08-07	KT16S408	16.100	35.173	174.591	0.000	0.000	-	0.000	0.433	-	-	WEC this study
2017-08-14	KT16S409	16.174	35.256	231.278	0.000	0.000	-	0.015	0.650	-	0.890	WEC this study
2017-08-21	KT16S410	17.698	35.295	233.601	0.015	0.000	-	0.000	0.818	-	0.970	WEC this study
2017-08-28	KT16S411	16.400	35.143	222.646	0.000	0.000	-	0.000	0.885	-	0.390	WEC this study
2017-09-04	KT16S412	15.213	34.678	220.830	0.000	0.000	-	0.035	1.168	-	0.900	WEC this study
2017-09-18	KT16S413	15.491	35.051	178.634	0.993	0.730	0.130	0.155	1.795	-	1.360	WEC this study
2017-09-25	KT16S414	15.567	35.028	225.872	1.950	0.750	0.105	0.185	2.700	-	1.140	WEC this study
2017-10-30	KT16S415	14.605	35.191	224.027	3.295	0.260	0.060	0.333	3.410	-	1.220	WEC this study
2017-11-13	KT16S416	14.048	35.256	225.475	3.575	0.105	0.030	0.348	3.195	-	0.920	WEC this study
2017-11-20	KT16S417	13.515	35.121	224.565	4.225	0.125	0.083	0.360	3.670	-	0.960	WEC this study
2017-12-04	KT16S418	12.526	35.199	232.053	4.447	0.098	0.130	0.380	3.770	-	0.830	WEC this study
2017-12-11	KT16S419	11.226	34.901	184.210	7.395	0.225	0.320	0.440	5.020	-	0.800	WEC this study
2017-12-18	KT16S420	10.752	34.792	241.412	7.345	0.225	0.127	0.438	4.775	-	0.710	WEC this study
2018-01-08	KT16S421	10.307	35.048	-	7.040	0.110	0.147	0.490	3.893	-	0.530	WEC this study
2018-01-22	KT16S422	9.970	34.681	-	8.433	0.160	0.228	0.503	4.010	-	0.440	WEC this study
2018-01-29	KT16S423	9.982	35.000	-	7.540	0.110	0.138	0.475	3.408	-	0.580	WEC this study
2018-02-05	KT16S424	9.608	34.974	236.476	7.260	0.135	0.170	0.483	3.418	-	0.510	WEC this study
2018-02-12	KT16S425	8.873	34.885	-	7.563	0.185	0.160	0.450	3.063	-	0.550	WEC this study
2018-02-19	KT16S426	9.125	34.270	-	11.287	0.270	0.453	0.453	4.653	-	1.290	WEC this study
2018-02-26	KT16S427	8.487	35.100	-	10.110	-	-	-	2.000	-	1.080	WEC this study
2018-03-05	KT16S428	7.787	35.046	232.564	6.938	0.248	0.207	0.453	3.148	-	0.420	WEC this study
2018-03-12	KT16S429	-	-	-	12.980	0.300	1.035	0.523	5.718	-	0.350	WEC this study
2018-03-19	KT16S430	7.849	34.647	243.424	8.678	0.235	0.385	0.475	3.715	-	0.950	WEC this study
2018-04-02	KT16S431	8.007	34.594	248.062	8.745	0.198	0.353	0.435	3.810	-	1.020	WEC this study
2018-04-09	KT16S432	8.366	34.622	244.833	15.668	0.275	0.360	0.355	6.413	-	3.950	WEC this study
2018-04-19	KT16S433	-	-	-	-	-	-	-	-	-	-	WEC this study
2018-04-26	KT16S434	-	-	-	-	-	-	-	-	-	-	WEC this study
2018-04-30	KT16S435	-	-	-	0.840	0.060	0.985	0.100	0.493	-	-	WEC this study
2018-05-07	KT16S436	11.559	35.064	-	0.025	0.000	0.080	0.035	0.338	-	1.450	WEC this study
2018-05-14	KT16S437	11.513	34.920	-	0.000	0.000	0.125	0.000	0.233	-	0.190	WEC this study
2018-05-21	KT16S438	11.818	35.188	-	0.000	0.000	0.053	0.068	0.475	-	0.380	WEC this study
2018-05-30	KT16S439	-	-	-	-	-	-	-	-	-	-	WEC this study
2018-06-04	KT16S440	14.526	35.150	-	0.000	0.000	0.000	0.015	0.330	-	0.250	WEC this study
2018-06-18	KT16S441	13.838	35.073	-	0.037	0.000	0.143	0.050	0.160	-	0.730	WEC this study
2018-06-25	KT16S442	15.283	35.150	-	0.020	0.000	0.115	0.040	0.073	-	0.350	WEC this study
2018-07-02	KT16S443	18.153	35.112	208.245	0.040	0.000	0.250	0.013	0.085	-	0.620	WEC this study
2018-07-09	KT16S444	18.148	35.115	207.566	0.033	0.000	0.128	0.033	0.063	-	0.290	WEC this study
2018-07-16	KT16S445	19.167	35.017	177.404	0.000	0.000	0.000	0.000	-	-	0.230	WEC this study
2018-07-23	KT16S446	16.853	35.097	201.752	0.000	0.000	0.000	0.030	-	-	0.590	WEC this study
2018-07-30	KT16S447	16.724	35.086	245.903	0.000	0.000	0.185	0.093	-	-	1.090	WEC this study
2018-08-06	KT16S448	17.884	34.964	265.454	0.020	0.000	0.250	0.020	0.568	-	0.380	WEC this study
2018-08-13	KT16S449	16.459	34.967	267.668	0.000	0.000	0.000	0.050	0.950	-	2.150	WEC this study
2018-08-20	KT16S450	16.471	34.966	266.252	0.015	0.000	0.440	0.040	1.613	-	1.050	WEC this study

2018-08-27	KT16S451	16.208	34.962	216.404	0.010	0.000	0.255	0.023	1.580	-	2.320	WEC this study
2018-09-03	KT16S452	16.675	35.040	244.267	0.050	0.020	0.755	0.135	1.700	-	0.640	WEC this study
2018-09-17	KT16S453	-	-	-	0.000	0.000	-	0.053	2.030	-	-	WEC this study
2018-12-03	KT16S454	12.117	34.628	253.638	7.393	0.243	0.305	0.445	4.398	-	0.760	WEC this study
2018-12-10	KT16S455	11.572	33.884	258.299	11.873	0.227	0.440	0.573	5.833	-	0.570	WEC this study
2018-12-17	KT16S456	-	-	-	8.525	0.188	0.388	0.493	4.558	-	-	WEC this study
